# Longitudinal clinical and proteomic diabetes signatures in women with a history of gestational diabetes

**DOI:** 10.1172/jci.insight.183213

**Published:** 2024-11-26

**Authors:** Heaseung Sophia Chung, Lawrence Middleton, Manik Garg, Ventzislava A. Hristova, Rick B. Vega, David Baker, Benjamin G. Challis, Dimitrios Vitsios, Sonja Hess, Kristina Wallenius, Agneta Holmäng, Ulrika Andersson-Hall

**Affiliations:** 1 Dynamic Omics, Centre for Genomics Research, Discovery Sciences, BioPharmaceuticals R&D, AstraZeneca, Gaithersburg, Maryland, USA.; 2 Centre for Genomics Research, Discovery Sciences, BioPharmaceuticals R&D, AstraZeneca, Cambridge, United Kingdom.; 3 Early Clinical Development, Early CVRM, BioPharmaceuticals R&D, AstraZeneca, Gaithersburg, Maryland, USA.; 4 Bioscience Metabolism and; 5 Translational Science and Experimental Medicine, Early CVRM, BioPharmaceuticals R&D, AstraZeneca, Cambridge, United Kingdom.; 6 Bioscience Metabolism, Early CVRM, BioPharmaceuticals R&D, AstraZeneca, Gothenburg, Sweden.; 7 Institute of Neuroscience and Physiology, Department of Physiology, Sahlgrenska Academy, University of Gothenburg, Gothenburg, Sweden.

**Keywords:** Metabolism, Diabetes, Proteomics

## Abstract

We characterized the longitudinal serum protein signatures of women 6 and 10 years after having gestational diabetes mellitus (GDM) to identify factors associated with the development of type 2 diabetes mellitus (T2D) and prediabetes in this at-risk post-GDM population, aiming to discover potential biomarkers for early diagnosis and prevention of T2D. Our study identified 75 T2D-associated serum proteins and 23 prediabetes-associated proteins, some of which were validated in an independent T2D cohort. Machine learning (ML) performed on the longitudinal proteomics highlighted protein signatures associated with progression to post-GDM diabetes. We also proposed prognostic biomarker candidates that were differentially regulated in healthy participants at 6 years postpartum who later progressed to having T2D. Our longitudinal study revealed T2D risk factors for post-GDM populations who are relatively young and healthy, providing insights for clinical decisions and early lifestyle interventions.

## Abstract 2

Our research reveals risk factors for women with prior GDM, who are relatively young and healthy but face a 10-fold higher risk of T2D.

## Graphical abstract

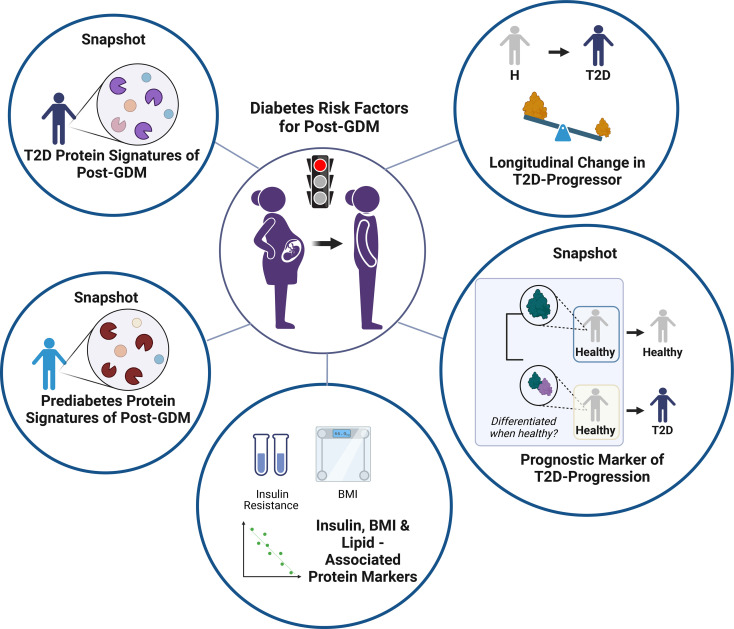

## Introduction

It is projected that over 700 million adults worldwide will have diabetes mellitus by 2045 (1), and this represents a considerable public health challenge. Effective prevention and treatment can substantially reduce the risk of complications associated with diabetes. Gestational diabetes mellitus (GDM), defined as glucose intolerance with onset or first recognition during pregnancy (2), has serious short- and long-term consequences for both women and their children. Even though normal glucose tolerance is typically restored after birth, recent meta-analysis concluded that women with a prior GDM diagnosis have as much as a 10-fold increased risk of developing type 2 diabetes mellitus (T2D) later in life (3). The absolute risk estimates for the development of T2D in women who had GDM can vary depending on ethnicity and GDM diagnosis criteria. For instance, a study in 2010 (4) reported that 30% of patients with GDM developed T2D after 5 years. A more recent international study, using the new GDM criteria, provides an accumulative estimate of 17% of patients with GDM (5). Consequently, the metabolic demands of pregnancy can identify a predisposition for T2D, and GDM represents one of the strongest risk factors for the development of T2D among young women (6, 7). With T2D being a preventable or delayable disease, there is an urgent need for precision screening and treatment (8). Moreover, onset of prediabetes and progression to T2D start years before clinical diagnosis, which makes this population of relatively young and healthy women with high risk of T2D particularly valuable for longitudinal studies aimed at investigating potential biomarkers as well as early pathophysiology of T2D.

Lifestyle changes have been shown to delay progression from prediabetes to T2D, but early diagnosis and intervention remain challenging (9, 10). This is particularly true for individuals with isolated impaired glucose tolerance (IGT), a substantial proportion of the prediabetes population, that can remain undetected by current T2D screening strategies measuring glycated hemoglobin (HbA1c) and fasting glucose levels (10). To identify patients with IGT early in their disease, it is necessary to perform a glucose challenge and measure postprandial glucose levels using an oral glucose-tolerance test (OGTT) (10). Currently, it is not clear if proteome features of participants who are prediabetic resemble those of T2D or if they display unique protein profiles specific to prediabetes.

Unbiased proteomics is well suited for finding biomarkers and indicators of risk for T2D onset and progression, given the polygenic nature of T2D with contributions from environmental and behavioral factors, which can affect proteins and metabolites (11). Therefore, there have been numerous studies examining proteomic and/or metabolic signatures of T2D or prediabetes across different ethnic populations (11–17). Similarly, plasma and serum proteomics of gestational diabetes samples obtained during pregnancy have been characterized previously in multiple cohorts (18, 19). However, to the best of our knowledge, a proteomics profile of post-GDM T2D has not been established.

In this study, we characterized proteomic signatures of mixed ethnicity women in Sweden (50% of participants not of Nordic descent) who were diagnosed with GDM as part of the Pregnancy Obesity Nutrition Child Health (PONCH) study (20, 21). The study included both matched and unmatched samples at 2 time points, 6 and 10 years postpartum. We first analyzed T2D- and prediabetes-associated serum proteins that correlated with clinical observations at either of the time points and validated the diabetic signatures by analysis of longitudinal changes in healthy participants at 6 years who went on to develop T2D or prediabetes at 10 years. Next, the clinical variables and protein levels of these participants were evaluated at 6 years to identify potential prognostic markers for T2D, which can aid in early diagnosis and management. Lastly, prediabetes-specific signatures were identified, which can provide better T2D risk stratification and improve early intervention and prevention of T2D.

## Results

### Post-GDM diabetic cohorts and their clinical characteristics.

**Figure 1:**
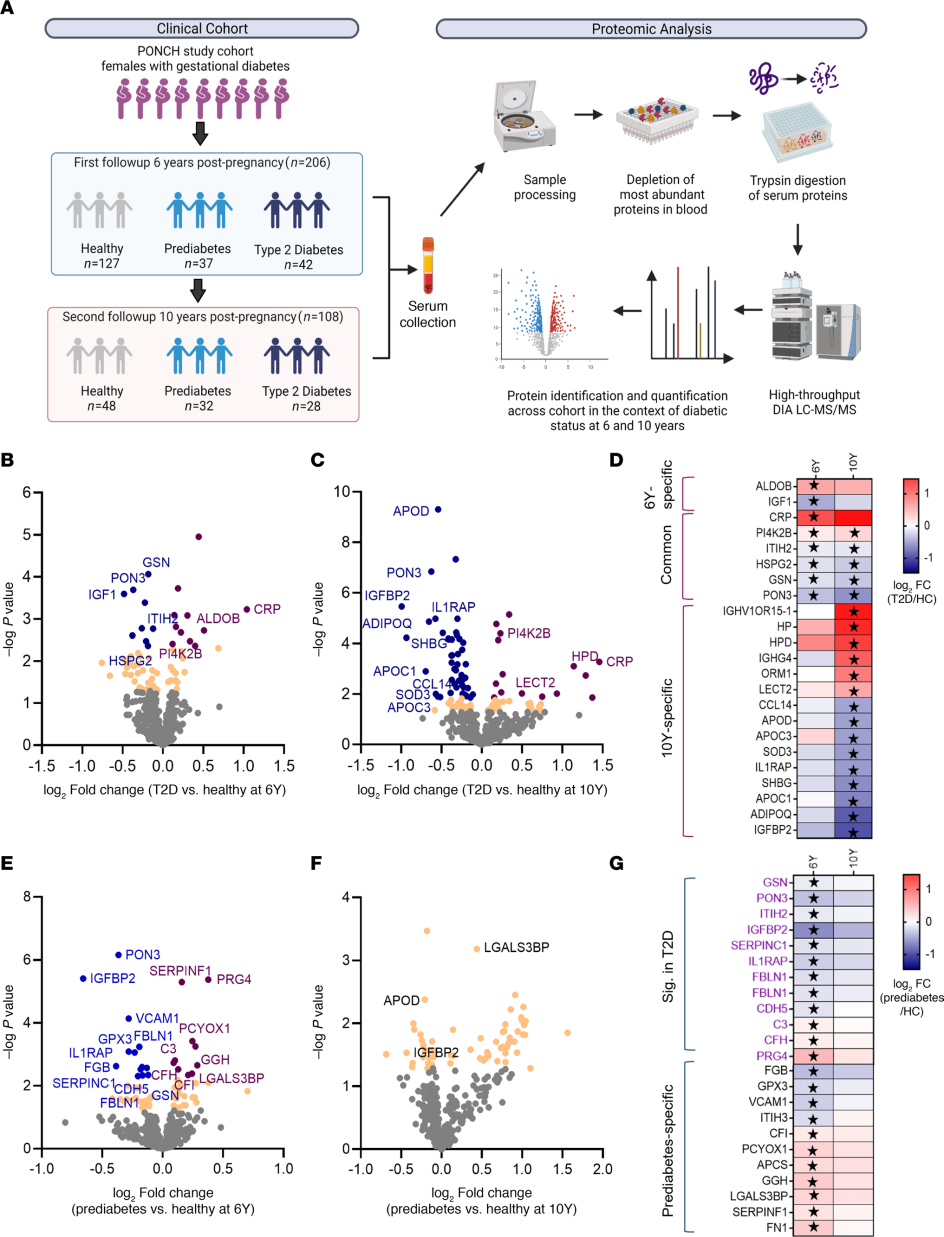
Comparisons of proteomes in T2D and prediabetes versus healthy participants. (**A**) Overview of clinical cohort and the serum proteomic workflow. Proteomic profiling was performed in serum samples collected at 6-year and 10-year postpregnancy follow-ups from participants diagnosed with GDM. Serum samples were prepared and analyzed by LC-MS/MS using data-independent acquisition (DIA). The total number of participants per subgroup is shown. (**B** and **C**) Volcano plots comparing the serum proteomes of T2D versus healthy participants at 6-year (**B**) and 10-year (**C**) follow-ups post-GDM. The log_2_ fold-change in protein abundance is displayed on the *x* axis, and the –log_10_ 2-tailed *t* test *P* value is displayed on the *y* axis. Color coding is based on *P* values (*P* < 0.05: in yellow), with directionality of difference in protein abundance (purple: significantly increased in T2D; blue: significantly decreased in T2D). Highlighted proteins are further discussed in the text. (**D**) Dysregulated proteins in T2D with the most pronounced magnitude of dysregulation (log_2_ fold change > 0.5) or observed at both 6- and 10-year visits, as shown in **B** and **C**. Proteins significantly dysregulated in T2D compared with healthy controls were marked with a star. (**E** and **F**) Volcano plots of prediabetic versus healthy participants proteome at 6-year (**E**) and 10-year (**F**) post-GDM visits. Notations are as in **B** and **C**. (**G**) Log_2_ fold change protein abundance in prediabetes compared with healthy participants at 6 and 10 years after GDM. Proteins found to be T2D associated in **B** or **C** are labeled purple and prediabetic-specific proteins are labeled black. Proteins significantly dysregulated in prediabetes compared with healthy controls were marked with a star.

In this study, serum samples were collected, and clinical measurements were taken from the PONCH cohort at 6- and 10-year follow-up visits after GDM pregnancy (20, 21). Participants were classified as normoglycemic (hereinafter referred to as “healthy”), prediabetic, or T2D. Diabetic status was determined at each follow-up visit by the WHO criteria for diagnosis of T2D (2) including fasting glucose, 2-hour OGTT glucose, and HbA1c or reporting of an existing diabetes diagnosis. The proteomics subcohort consisted of 213 participants who contributed samples for analysis on at least one of the follow-up visits (Figure 1A and Supplemental Figure 1A; supplemental material available online with this article; https://doi.org/10.1172/jci.insight.183213DS1). At the 6-year visit, 206 individuals participated, and of these, 127 were categorized as healthy, 37 as prediabetic, and 42 as having T2D. Of the participants with T2D at the 6-year visit, 8 (19%) were treated with metformin and 3 (7%) with insulin. Twenty-two of 42 (52%) participants with T2D were self-reported, while 20 (48%) were newly diagnosed through their study participation (Supplemental Figure 1B). At the 10-year visit, 108 participants were screened, and 48, 32, and 28 participants were classified as healthy, prediabetic, and having T2D, respectively. Among the 28 participants with T2D at 10 years, 13 (46% of the 28) were being treated with metformin and 3 (11%) with insulin. At 10 years, 17 of 28 (61%) participants with T2D were self-reported, while 11 (39%) were newly diagnosed (Supplemental Figure 1B). Additionally, 21 (66%) of the participants who were prediabetic at the 10-year visit were newly diagnosed as prediabetic. Importantly, the drop-out analysis indicated no selection bias between women included in the 6-year analysis only and those included in both the 6- and 10-year analyses (Supplemental Methods). In a cross-sectional analysis, participants who subsequently developed prediabetes or T2D showed enhanced metabolic risk factors during GDM pregnancy (Table 1). These factors included obesity (BMI ≥ 30 kg/m^2^) at the start of the index pregnancy, earlier GDM diagnosis (end of second trimester versus beginning of third trimester), and severe GDM requiring insulin treatment. Additionally, women with post-GDM T2D had increased insulin-resistance index HOMA-IR, circulating lipids, leptin, and blood pressure. Notably, half of the women who developed post-GDM T2D were not clinically obese (BMI < 30 kg/m^2^) (22) but were of normal weight (18%–19%) or overweight (36%–38%) (Table 1). The participants who developed T2D also displayed signs of low-grade systemic inflammation (elevated C-reactive protein [CRP]) compared with healthy women at both 6 and 10 years. In contrast, adiponectin levels were lower in T2D than healthy women.

### Proteomics detected T2D-associated proteins in post-GDM population.

To comprehensively analyze the serum proteome of these post-GDM women focusing on different diabetic states, we used an unbiased and robust liquid chromatography–tandem mass spectrometry–based (LC-MS/MS–based) proteomics workflow using a data-independent acquisition (DIA) method (Figure 1A). This approach allowed us to identify and quantify over 400 protein groups spanning more than 4 orders of magnitude of abundance in serum (Supplemental Figure 1C). Prior to comparative analysis, we evaluated the precision of our quantification by examining technical reproducibility (Supplemental Figure 1D). Additionally, we ensured the robustness of our method by a tight correlation between protein quantity values measured by LC-MS/MS and ELISA of CRP (ρ = 0.98) and adiponectin (ρ = 0.63), which are established inflammatory and antiinflammatory markers in T2D (23, 24) (Supplemental Figure 1E).

We investigated differences in serum proteome of participants with T2D versus healthy controls from the 6-year-visit and 10-year-visit cohorts by cross-sectional analysis. In the 6-year-visit cohorts, 20 differentially expressed proteins were identified, including 9 proteins with decreased levels and 11 proteins with increased levels in T2D (Figure 1B). In the 10-year-visit cohorts, we found a total of 61 significantly differentially abundant proteins in T2D compared with healthy controls (Figure 1C), with 46 (75%) being downregulated and 15 (25%) upregulated. Since HOMA-IR of women with T2D increased at 10 years (median, 5.1) compared with 6 years (median, 3.6), this implies further T2D disease progression at the 10-year visit compared with the 6-year visit (Table 1), and this could explain the increase in differentially abundant proteins in individuals with T2D versus healthy individuals at 10 years. We identified 6 common proteins that were differently expressed in T2D serum compared with the healthy controls at both time points, representing T2D-associated protein signatures that are independent of duration of diabetes and may serve as potential markers for both early and late T2D (Figure 1D). Other proteins that reached statistical significance at the second visit exhibited similar fold-change trends at both time points (Figure 1D). The 75 T2D-associated proteins from either of the cross-sectional analyses (Supplemental Data File 1) included established T2D signatures previously reported, such as insulin-like growth factor-binding protein 2 (IGFBP2) or sex hormone-binding globulin (SHBG), but also included relatively new T2D-associations with proteins such as heparan sulfate proteoglycan 2 (HSPG2). We did not identify any significantly enriched pathways among the 75 proteins associated with T2D by Ingenuity Pathway Analysis (IPA) and Fisher’s exact test (*q* > 0.05). Additionally, we performed a first sensitivity analysis, adjusting for age and BMI. From the analysis, we observed consistent trends in 80% and 89% of the T2D protein signatures at 6 and 10 years, respectively (Supplemental Figure 2, A and B, and Supplemental Data File 2). Following this, we ran the second model to additionally adjust for metformin and insulin treatment. After this adjustment, 14 of 20 proteins at 6 years and 40 of 61 proteins at 10 years still held the same trend (*P* < 0.05), including CRP, GSN, HSPG2, and PON3 (Supplemental Data File 2).

Excess glucose in the bloodstream has long been known to undergo a chemical reaction with lysine residues in proteins, initially forming a Schiff’s base that subsequently converts into an Amadori product (a ketoamine or fructosamine) (25, 26). This is referred to as nonenzymatic glycation and is clinically exploited for HbA1c measurement to monitor glycemic control (27, 28). Both glycated albumin and fructosamine on other circulating proteins can be measured for assessment of hyperglycemia in settings where HbA1c testing is not available (28, 29).

Taking advantage of the power of our proteomics approach, we examined nonenzymatic glycation events in T2D. In the approach, we used a direct measurement of glycated peptides. We identified over 100 glycated peptides, with hemopexin and albumin peptides exhibiting higher glycation levels in T2D compared with healthy controls from the 6- and 10-year cohort (Supplemental Data File 3), consistent with previous reports (25, 30, 31). Notably, while the glycated peptide levels were higher in T2D, the protein expression levels of hemopexin and albumin were not significantly different in participants with T2D compared with healthy controls. This analysis suggests an elevation in glycation levels in T2D, confirming the power of posttranslational analysis in gaining additional clinical insights.

### Correlation of diabetes-associated proteins with clinical observations.

Next, we investigated whether the proteome of participants with prediabetes displayed similar, milder, or different signatures from those observed in the T2D cohort. Cross-sectional analysis at 6 years identified 23 proteins that were significantly differentially abundant in prediabetes compared with healthy controls, with 10 proteins up- and 13 downregulated (Figure 1E). Differential expression in prediabetes at 10 years was less extensive, not reaching statistical significance (Figure 1F). This could be, in part, attributed to less advanced prediabetes in these participants, as 60% of them were healthy 4 years prior, at the 6-year time point (Supplemental Figure 2, C and D). Alternatively, this may be due to the statistical power and smaller sample size of the healthy controls at the 10-year visit (Table 1). Importantly, most of the 23 prediabetes-associated proteins followed the same trend at both time points (Figure 1G).

Of these 23 proteins, 12 were also associated with post-GDM T2D, including IGFBP2, PON3, IL1RAP, and complement proteins (CFH and C3) (Figure 1, B, C, and G). The remaining 11 proteins were associated only with prediabetes and included vascular cell adhesion protein 1 (VCAM1) and galectin-3-binding protein (LGALS3BP). Increased levels of LGALS3BP were observed in prediabetes at both time points (*P* < 0.05), and the association held after adjustment for age and BMI in both cross-sectional prediabetes versus healthy comparisons (Supplemental Figure 2, E and F). Adiponectin (ADIPOQ) was associated with T2D but was not changed in the prediabetes population (Supplemental Data File 1).

**Figure 2:**
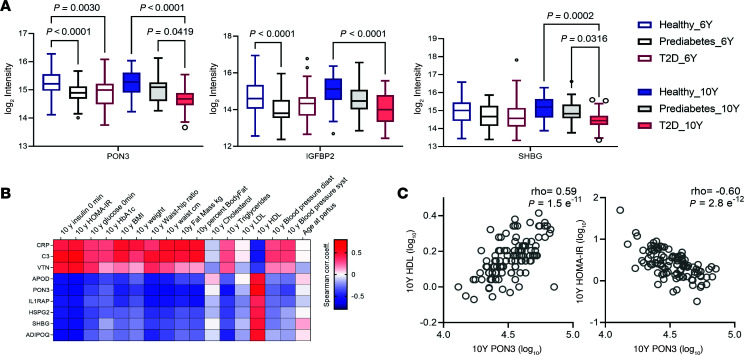
Proteins associated with diabetes severity or clinical traits. (**A**) Intensities of the selected proteins (1-way ANOVA, *P*_adj._ <0.01) across subpopulations. Log_2_ intensity of participants in each subpopulation were displayed as a box-and-whiskers plot showing median and interquartile range (IQR). Adjusted *P* value by Brown-Forsythe and Welch 1-way ANOVA tests and Dunnett’s T3 multiple comparisons test were labeled if *P* < 0.05. (**B**) Associations of post-GDM T2D markers from Figure 1, B and C, with clinical characteristics. Selected proteins with absolute Spearman correlation ρ > 0.4 with at least 1 of the clinical characteristics were displayed. Heatmaps show correlation coefficients between protein levels in serum and clinical characteristics of all participants at 10 years follow-up visits. Row clustering was based on log_2_ intensity of the protein. (**C**) Examples of the correlations from **B**; abundance of PON3 protein with HDL and insulin resistance of all participants at 10-year time point.

Additionally, the abundance of these proteins was found to correlate with diabetic severity. One-way ANOVA tests across participant groups showed protein abundance trends aligned with diabetic status and progression (Figure 2A, Supplemental Figure 3, and Supplemental Data File 4). Specifically, PON3, SHBG, and IGFBP2 were further downregulated as the diabetic status became more severe (Figure 2A). Differentially expressed proteins were highly correlated with insulin, body weight, and lipid-related clinical parameters at both time points (Figure 2, B and C).

### Longitudinal analyses of BMI and insulin resistance in diabetes onset.

**Figure 3:**
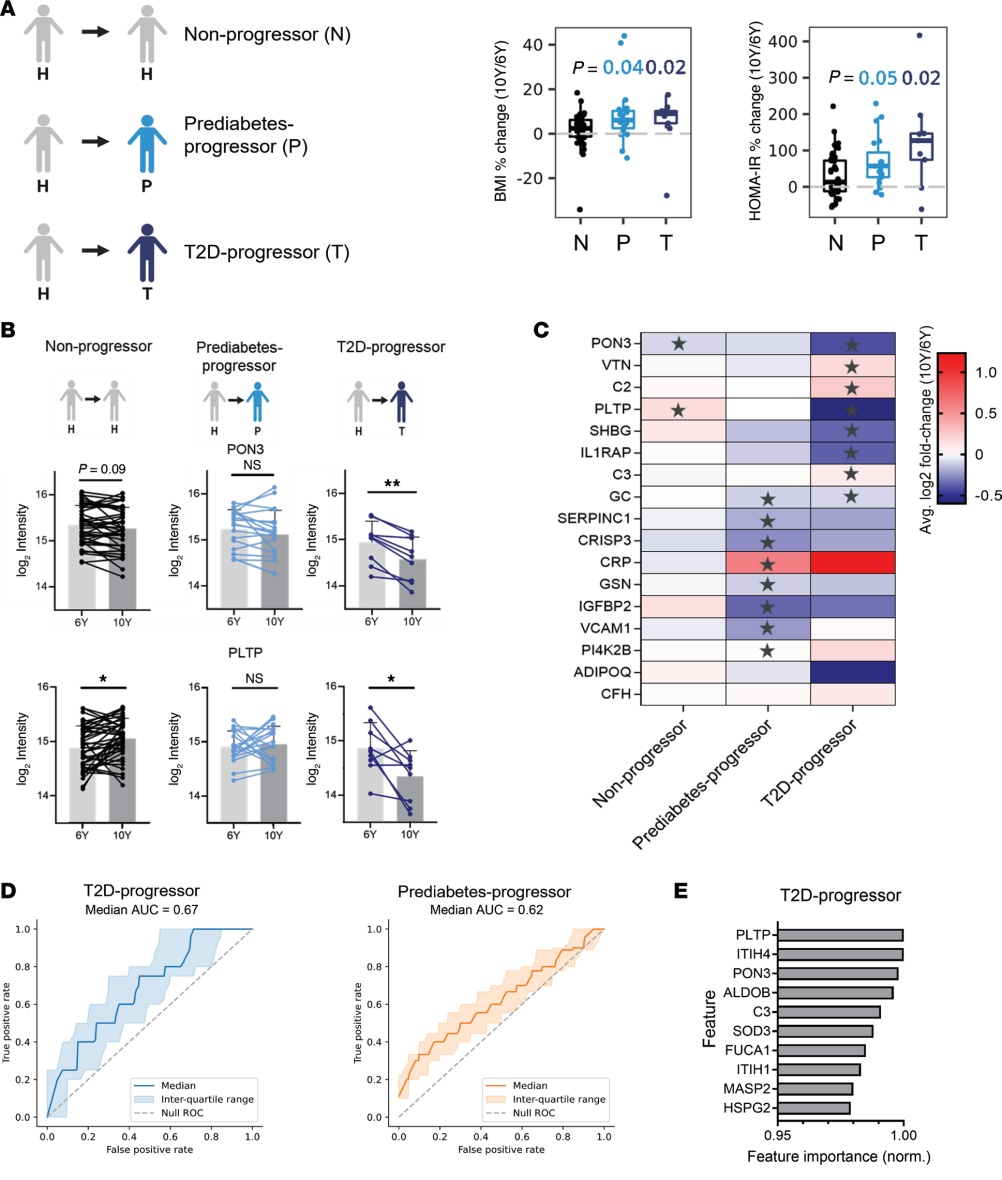
Biomarker candidates of diabetes progression in longitudinal analysis. (**A**) Left: Overview of clinical cohorts for clinical and serum proteomic longitudinal analyses. Among the healthy participants at the 6-year visit, subpopulations who developed either prediabetes (*n* = 19) or T2D (*n* = 9) were labeled as the diabetes-progressors, while nonprogressors stayed healthy at the 10-year visit (*n* = 41). Right: Progression to diabetes between 6-year and 10-year follow-up visits was associated with increase in BMI and HOMA-IR. The plots show within-participant changes in BMI (top) and HOMA-IR (bottom) with Wilcoxon *P* values of progressors versus nonprogressors. (**B**) Protein changes at 10 years versus 6 years within a participant across each subcohorts are represented with individual lines. PON3 (top) and PLTP (bottom) changed most in T2D-progressors between 6 and 10 years. Data represent mean ± SD of each year. *P_adj._ < 0.05, **P_adj._ < 0.01. (**C**) Protein change between 6 and 10 years within a participant across each subcohort is displayed in colors corresponding to the mean of the log_2_ fold change (10 years/6 years) within a participant. Among the T2D or prediabetes-specific proteins from Figure 1, proteins mostly changed in T2D-progressor or prediabetes-progressor are shown (Supplemental Data File 5). A star is displayed in each cell if abundance of a protein was significantly changed at 10 years compared with 6 years by paired 2-tailed *t* test. (**D**) ROC curve and corresponding AUC statistics using random forest model, using a 2-fold stratified cross-validation and repeated process over 5,000 within-class shuffling to differentiate T2D-progressors and prediabetes-progressors from nonprogressors. (**E**) The 10 most discriminating features of T2D-progressors versus nonprogressors for model training.

In addition to the cross-sectional analysis, we conducted longitudinal analysis to investigate the progression of diabetes in this cohort. Healthy participants were stratified at 6 years based on their future outcome. Among the 69 participants who were healthy at the 6-year visit, 41 participants (59%) remained healthy at the 10-year follow-up (hereinafter referred to as nonprogressors), 19 participants (28%) progressed to prediabetes (prediabetes-progressors) and 9 participants (13%) developed T2D (T2D-progressors; Figure 3A).

Longitudinal analysis found that prediabetes-progressors and T2D-progressors had a higher percent increase in BMI and HOMA-IR between the 6-year and 10-year visits compared with nonprogressors (Figure 3A). Within-individual changes in other clinical parameters also correlated with weight gain and worsening HOMA-IR (Supplemental Figure 4A).

To ensure that the observed changes were specific to diabetes, we assessed related comorbidities based on circulating levels of cardiovascular (32, 33), kidney filtration (34), and endothelial dysfunction biomarkers (35) measured with the untargeted DIA proteomics method and found that these did not change in participants whose condition progressed to prediabetes or T2D (Supplemental Figure 4, B–D), further confirming that the observed clinical trends were diabetes specific.

### Longitudinal proteomic changes in disease progression.

Next, we investigated how the proteome changed over time within individual participants and whether these changes could distinguish between progressors and nonprogressors (Figure 3, B and C). One hundred and one participants provided serum for proteomics at both time points, and of these, paired samples from 69 healthy participants at 6 years were evaluated by comparing fold changes of protein abundance between the 6- and 10-year visit. Thirteen proteins showed significant changes over the 4-year period in the T2D-progressors regardless of BMI adjustment (Supplemental Data Files 5 and 6). These proteins included PON3 and phospholipid transfer protein (PLTP), which were previously identified as associated with T2D in our study (Figure 1 and Supplemental Data File 1). PON3 showed a greater tendency to decline over the 4 years between the 6- and 10-year visits in the T2D-progressor group (paired adjusted *P* [*P*_adj._] ≤ 0.01 and log_2_[10 years/6 years]: –0.42) compared with the nonprogressor group (paired *P*_adj._ = 0.09 and log_2_[10 years/6 years]: –0.1) (Figure 3B). We compared the 75 T2D- and 23 prediabetes-associated signatures from the larger-group unpaired analysis (Figure 1 and Supplemental Data File 1) with the progression-associated proteins from the paired samples of progressors. PON3 exhibited a sharp decline in participants with T2D or prediabetes at both visits (Figure 2A). The same directional changes in T2D or prediabetes-progression were confirmed in a subset of proteins listed in Figure 3C (*P* < 0.05).

Furthermore, we compared cross-sectional analyses of the prediabetes population with the prediabetes progression-associated proteins and confirmed the matched prediabetic changes. For example, VCAM1 declined between 6 and 10 years exclusively in the prediabetes-progressor subpopulation (Figure 3C), and this decline matched the downregulation of VCAM1 in the larger group of prediabetes (Figure 1G). Interestingly, LGALS3BP, which was prediabetes associated (Figure 1G), showed an increase over time in T2D-progressors but not in prediabetes-progressors or nonprogressors (Supplemental Data File 5).

### Protein signature inference with supervised machine learning.

We next conducted supervised machine learning (ML) analyses to infer protein signatures associated with T2D progression (Supplemental Figure 5). Initially, we assessed logistic regression, gradient boosting, and random forest models using only proteomic data. We employed (shuffled) stratified k-fold cross-validation (k = 2) to estimate the area under the curve (AUC) and repeated the process over 5,000 times within-class shuffling, to ensure robustness in predictions. Random forest emerged as the top performer based on AUC classification, leading us to select it with 100 estimators to model both proteomic and clinical covariates, including BMI, blood pressure, HDL, and LDL (Supplemental Figure 5B). HOMA-IR, insulin, glucose level, and HbA1c were not included because these measurements are routinely used in clinical practice for diabetes diagnosis. The final model incorporated 49 clinical covariates, yielding a total of 477 features, including 428 proteins.

We achieved an acceptable level (36) of predictive performance across proteomic and clinical covariate datasets, with a median AUC of the ROC (receiver operating characteristic) curve of 0.67 (Figure 3D). To identify the features important for predicting T2D-progressors from nonprogressors, we used a permutation feature importance method. We computed the final measure of feature importance by averaging 200 random forests, aiming to reduce variability. The most discriminating proteins or clinical indices for model training were then selected and ranked based on their feature importance (Figure 3E). Notably, proteins such as PLTP and PON3, previously highlighted in Figure 3D, emerged among the most T2D progression–associated proteins. Additionally, ITIH4, C3, and SOD3, which distinguished T2D-progressors compared with nonprogressors in our earlier analysis, emerged as promising candidates for classification (Supplemental Data File 5). Using the same ML approach, we identified the relative importance of features predictive of prediabetes-progressors (Figure 3D and Supplemental Figure 5D). Among the top 10 features, 6 were clinical covariates such as fat mass, waist, and BMI and 4 were protein features including FCN2 and TGF-β1 as shown in Supplemental Data File 5.

To independently validate our findings, we performed a correlation analysis using UK biobank pQTL studies/proteomics studies and associated public health records. There was a significant correlation between the importance of the ML features in the PONCH study and statistics derived using independent UK Biobank data (37, 38) in a relevant cohort for T2D-progressors (*r* = 0.152, *P* = 0.023; Supplemental Figure 5F). There was no significance detected for the patients progressing to prediabetes. This could be due to the validation cohort not being enriched for prediabetes (Supplemental Methods). Furthermore, in our earlier analysis, AUC for the prediabetes-progressor was less discriminative. In summary, our proteomics data, coupled with ML algorithms, effectively discriminated T2D-progressors from nonprogressors, reinforcing the significance of T2D progression–associated proteins and suggesting potential clinical applications for enhancing diagnostic accuracy.

### Potential prognostic marker candidates in post-GDM women.

**Figure 4:**
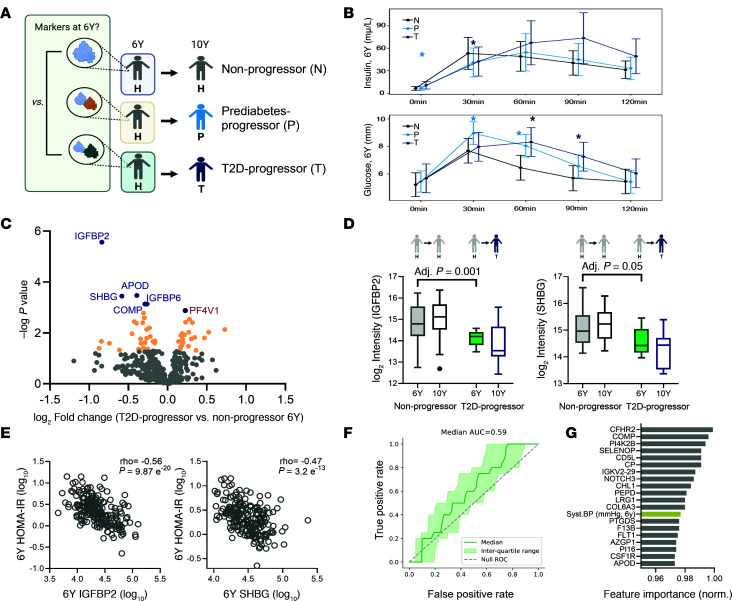
Differential expression of nonprogressor versus T2D-progressor at 6-year visit, when subcohorts were healthy. (**A**) Subcohorts overview for prognostic signature discovery. (**B**) Response to OGTT in healthy participants at 6 years showed early signs of their 10-year outcome. Data are shown as mean ± SD levels over time after the oral glucose challenge based on subgroup. Due to limited sample size, this analysis was performed on all participants who had 6-year OGTT data and known outcome at 10 years, and it was not restricted to participants who contributed serum for proteomics. *N* healthy at both 6 and 10 years (“H”, nonprogressor) = 51, *N* healthy at 6 years who progressed to prediabetes at 10 years (“P”, prediabetes-progressor) = 24, *N* healthy at 6 years who progressed to T2D at 10 years (“T”, T2D-progressors) = 11. Significant differences between progressors and nonprogressors at specific time points after OGTT (*P* < 0.05) is denoted with an asterisk. (**C**) Volcano plot displaying differential protein expression in healthy to T2D-progressors (*n* = 9) versus participants staying healthy at 6-year visit (*n* = 41). The log_2_ fold change in protein level is displayed on the *x* axis, and the –log_10_ Welch’s 2-tailed *t* test *P* value is displayed on the *y* axis. Color coding is based on *P* values (*P* < 0.05: in yellow), with directionality of difference in protein abundance (purple: significantly increased; blue: decreased in T2D-progressors). Significantly different proteins are highlighted and further discussed in the text. (**D**) IGFBP2 and SHBG protein change at 10 years versus 6 years; Left: Healthy nonprogressors at both time points. Right: Healthy to T2D-progressors. Log_2_ intensity of participants in each subpopulation were displayed as a box-and-whisker plot showing median and IQR (Tukey method). (**E**) IGFBP2 and SHBG protein abundance with corresponding insulin resistance of all participants at the 6-year time point. Spearman correlation coefficient and *P* value were displayed. (**F**) ROC curve and corresponding AUC statistics to differentiate T2D-prognostic features. (**G**) The 10 most discriminating features as T2D-prognostic marker candidates.

Next, our analysis aimed to identify signatures that could predict the progression of T2D in participants who previously had GDM (Figure 4A). To do this, clinical and proteomic features of healthy participants at the 6-year visit were further investigated. At the 6-year visit, when participants were healthy, T2D-progressors of the participants tended to have higher BMI (nonsignificant trend) and HOMA-IR and lower thyroid-stimulating hormone (TSH) than nonprogressors (Supplemental Data File 7). By contrast, levels of leptin, ADIPOQ, and CRP did not differ between the subcohorts (Supplemental Data File 7). Notably, T2D-progressors started showing abnormalities in the shape of the OGTT curve already at the 6-year visit (Figure 4B). The peak in serum insulin and glucose tended to occur later after oral glucose administration in T2D-progressors compared with nonprogressors.

From the proteome perspective, we investigated proteins that were already differentiated at the 6-year visit in T2D-progressors versus nonprogressors as prognostic marker candidates of T2D. The analysis showed that 6 proteins, SHBG, APOD, IGFBP2, IGFBP6, cartilage oligomeric matrix protein (COMP), and platelet factor 4 variant 1 (PF4V1), were differentially expressed when both T2D-progressors and nonprogressors were healthy (Figure 4C). Low levels of IGFBP2, IGFBP6, APOD, and COMP support the previous observation that there was no significant decline in the proteins at the 6- versus 10-year time points within an individual of the T2D-progressors (Supplemental Data File 5) because the protein level was already low. Similarly, those proteins were expressed to a low degree in the larger T2D population compared with the healthy population at the 10-year comparison (Figure 1D and Supplemental Data File 1). Notably SHBG was already low in the T2D-progressors, and its level further declined over time (Figure 4D and Supplemental Figure 6B). When the participants were healthy, a low level of IGFBP2 was found in the T2D-progressors at the 6-year time point, and IGFBP2 was maintained low at the 10-year time point (Figure 4D). T2D-progressors preserved lower levels of the 5 downregulated proteins listed above, at the 10-year time point, similar to the subcohorts of patients who were diagnosed with T2D at both time points (Supplemental Figure 6, A and C).

After adjustment for age and BMI at the 6-year visit, 36 proteins were found to be nominally associated with progression to T2D (*P* < 0.05) (Supplemental Figure 6D). These proteins included the previously discussed 6 prognostic marker candidates such as IGFBP6 and, additionally, peptidase D (PEPD) and prostaglandin-H2 D-isomerase (PTGDS). Further analysis comparing nonobese T2D-progressors (*n* = 5) with the nonprogressors (*n* = 41) showed that the dysregulation of the 6 proteins was sustained with SHBG, IGFBP2, and APOD (*P* < 0.05) (Supplemental Figure 6E). This suggests that the protein signatures, along with insulin resistance, could be used as T2D risk factors for post-GDM women. Among the prognostic marker candidates, SHBG and IGFBP2 tightly correlated with insulin resistance, while others displayed a weaker correlation (Figure 4E and Supplemental Figure 6F).

We then used ML once more to distinguish between T2D-progressors and nonprogressors, this time focusing solely on protein information from the 6-year visit (and data prior to the visit). We observed an intriguing AUC of 0.59 and strong ROC curves demonstrating that there is predictive power in the dataset, despite the long time horizon (Figure 4F). Using the random forest model, as previously described, we identified the most significant features as potential prognostic markers (Figure 4G and Supplemental Figure 5). COMP, APOD, PEPD, and PTGDS emerged as top features, bolstering the confidence in these proteins as potential prognostic markers for T2D. Furthermore, while the primary purpose of the classification model was to gain insight into which proteins were driving the clinical outcomes through their feature importance, we noted the ROC AUC above the baseline 0.5 in all cases (median AUC larger than 0.55 in all cases and > 0.6 in 6 of 9 models; Supplemental Figure 5E), suggesting that there is predictive value in the dataset, albeit with an AUC that is not as high as in another related study in 2020 (39). Indeed, we noted that the inclusion of proteomic data significantly increased the predictive performance (*P* = 4.49 × 10^–7^) for the T2D-progressor model (Supplemental Figure 5E). Significance was quantified using the 1-sided Mann-Whitney *U* test over the distribution of AUCs obtained through multiple shuffled k-fold validations.

Furthermore, we investigated potential prognostic markers for prediabetes by analyzing the differential expression of proteins in prediabetes-progressors at 6 years (Supplemental Figure 7, A–C). Before and after adjustment for age and BMI at the 6-year visit, nominal associations were found with progression to prediabetes (*P* < 0.05) for 29 and 45 proteins, respectively, although these differences did not reach statistical significance with FDR adjustment (Supplemental Figure 7, B and C).

### Generalizability of post-GDM T2D associations across general T2D populations.

**Figure 5:**
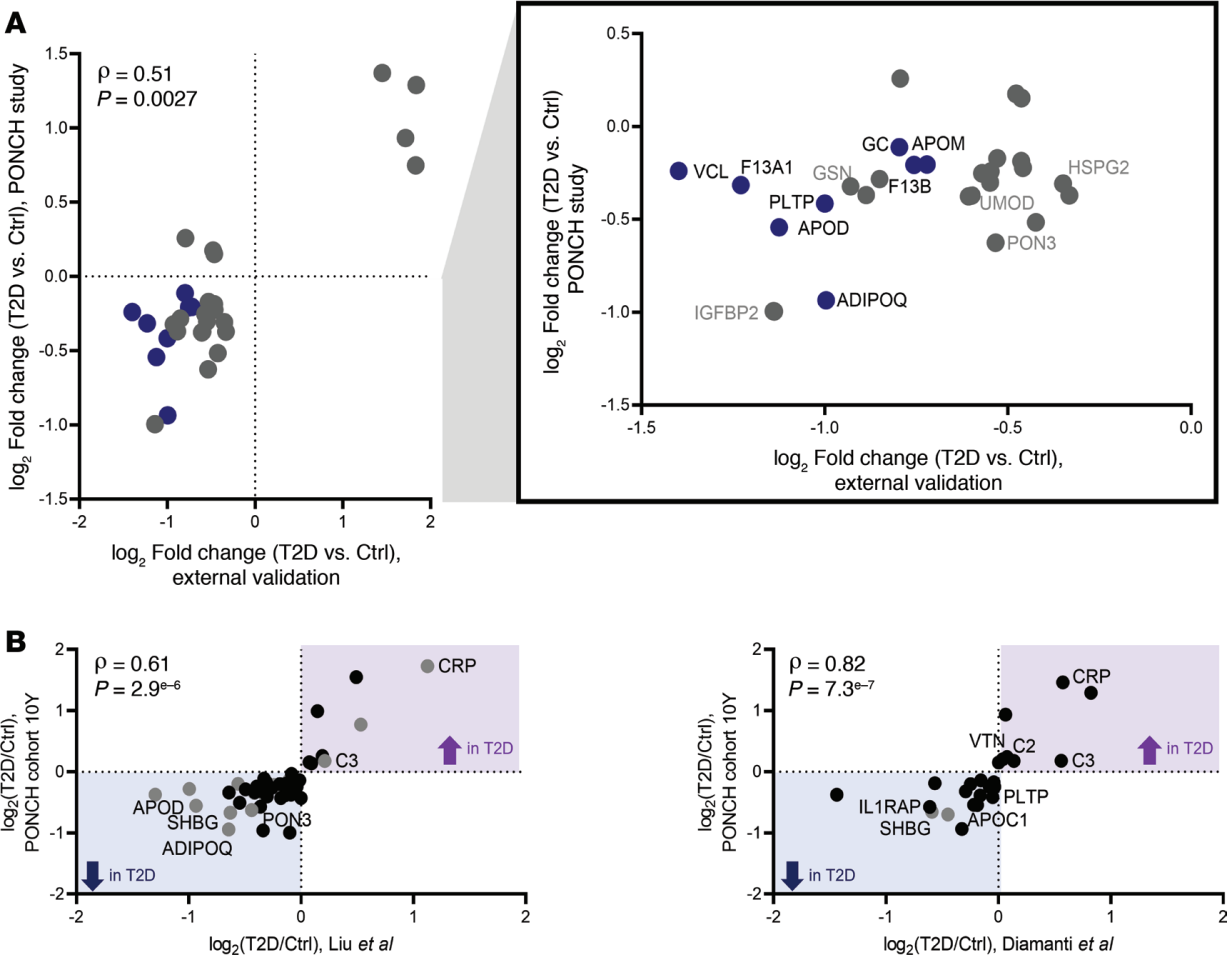
Comparison and validation of PONCH findings with external studies. (**A**) Correlation of T2D-associated proteins in PONCH study and corresponding proteins from validation cohort (Benjamini-Hochberg FDR < 30%). Spearman correlation coefficient and *P* value displayed tight correlation between the proteins from 2 independent studies. Data point of proteins downregulated in T2D external cohort (Benjamini-Hochberg FDR < 15%) were in blue and labeled in bold. Characteristics of the cohorts are presented in Table 2. (**B**) Strong correlation between the proteins from PONCH study versus 2 independent studies. Log_2_ fold-changes (T2D/Ctrl) for T2D-associated proteins identified in the PONCH 10-year cohort and reported by Liu et al. (left) (40), and Diamanti et al. (right) (41). Left: Forty-nine proteins common across the 2 studies with the same directional change in T2D, highlighting those that increase with T2D (purple) and those that decrease (blue). Right: Comparison with Diamanti et al. Twenty-four proteins decreased/increased in a mixed sex subcohort of Diamanti et al. as observed in the PONCH cohort at 10 years. Data point of significantly changed proteins in Liu et al. were labeled in gray (*P* < 0.05). Signature proteins are highlighted in Table 3; CRP and ADIPOQ were labeled with their names. Spearman correlation coefficient and *P* value displayed. Characteristics of the cohorts are presented in Table 2.

Next, we investigated if the biomarkers observed in the unique post-GDM PONCH cohort could be extrapolated to general T2D populations (Table 2 and Figure 5). An external validation cohort consisted of general T2D and healthy controls of White but non-Swedish female participants with unknown history of GDM. Including both premenopausal (29 participants, <50 years) and postmenopausal females (27 participants, ≥50 years). No participants had smoking history, and 20 (83%) of the participants with T2D were medicated with insulin. Twelve (50%) of the participants with T2D had comorbid hypertension, and 8 (33%) had comorbid atherosclerotic cardiovascular disease. We processed and analyzed serum samples from this cohort via the identical proteomics platform used for the PONCH study (Supplemental Figure 8A). Of the 75 T2D-associated serum proteins from the post-GDM PONCH study (Figure 1), 37 proteins showed a T2D association with a Benjamini-Hochberg FDR of 30% or lower. The consistent directional change was observed in 84% of the proteins (31 of 37 proteins) in the general T2D validation cohort (Supplemental Data File 8). Fold-changes of the 37 T2D-associated proteins correlated between the 2 independent cohorts (ρ = 0.51, *P* = 0.0027), post-GDM T2D and general T2D (Figure 5A).

We then further compared the findings from the PONCH study with results from publicly available T2D studies. Liu et al. (40) analyzed serum proteomes from both healthy controls and individuals with T2D with duration of clinically diagnosed diabetes of >10 years. The participants comprised both males and females, with an average BMI of 25.3 ± 3.4 and an average age of 54.9 ± 10.3 years in the T2D group. Among the 49 T2D-associated protein signatures observed in the PONCH cohorts at 10-year follow-up, 18 proteins were significantly changed in Liu et al. T2D cohorts, including SHBG, APOD, and C3 (Figure 5B and Supplemental Figure 8B). The fold changes of these T2D-associated proteins exhibited a strong correlation between the 2 independent studies (ρ = 0.61, *P* = 2.9 × 10^–6^), suggesting that a subset of T2D signatures could be applicable to an older, mixed-sex population. We further assessed the candidate biomarkers using serum proteins from another study (41). In a T2D subcohort of Diamanti et al., which consisted of an older, mixed-sex population, the differential expression of serum SHBG, APOC1, C3, and L-selectin (SELL) proteins was replicated, as observed in the PONCH study cohorts (Figure 5B). In the same study by Diamanti et al., we conducted additional investigations within a smaller subcohort consisting of postmenopausal women, 7 participants without diabetes, and 3 participants with T2D, confirming lower SHBG levels in the postmenopausal women with T2D (Supplemental Figure 8C).

Subsequently, we systematically assessed publicly available literature and ML approaches for the 15 key proteins summarized in Supplemental Table 1. The literature review revealed T2D or prediabetes associations with 10 proteins, including PON3, PLTP, and SHBG. To the best of our knowledge, there is no previous literature claiming a direct T2D (or prediabetes) association for the following 5 proteins: IL1RAP, IGFBP6, APOD, COMP, and FCN2, although a few sources suggest associations with weight, obesity, inflammation, or a role in adipose tissue (42–48). Finally, we observed support for PON3, APOD, IL1RAP, VTN, VCAM1, and IGFBP2 genes/proteins associated with T2D (top 5% in a framework) using an orthogonal and comprehensive ML framework (49–51), leveraging thousands of publicly available annotation data, including UK Biobank’s health record data.

## Discussion

Obesity is identified as a key risk factor for development of T2D in the general population (52–54), and weight loss is often proposed as a primary treatment goal for diabetes reversal among individuals with obesity (52–54). However, post-GDM T2D presents in relatively young and healthy women, who may not necessarily exhibit obesity. In our PONCH study, 36%–38% and 18%–19% of individuals with post-GDM T2D were overweight (BMI = 25–30 kg/m^2^) or normal weight (BMI = 18.5–25 kg/m^2^), respectively, but not clinically obese (BMI ≥ 30 kg/m^2^) at both 6- and 10-year visits (Table 1), suggesting that weight may not be the sole risk factor in this specific population.

It remains unclear whether T2D in women with a history of GDM shares the same pathobiology and features as T2D in the general population. Given that most women with GDM revert to normoglycemia after giving birth but have a 10× higher risk of developing T2D later in life (3, 5), and considering that T2D is a preventable but often silent disease, it is crucial to understand the pathophysiology of T2D after GDM and its prognostic factors.

Our study identified signature proteins that are highly associated with insulin resistance and progression to diabetic status using state-of-the-art proteomics techniques (55) (Table 3). The uniqueness of our study lies in its longitudinal design, offering profound insights into disease severity and progression reflected in proteomic shifts, as well as offering first-in-class protein signatures of T2D and prediabetes in this distinct population of post-GDM women. Moreover, our study cohort was meticulously controlled, minimizing confounding variables that often obscure genuine risk factors. Common T2D confounding diseases, as assessed by markers of cardiovascular risk and renal dysfunction, were rare. From a technical perspective, the unbiased proteomics analysis employed in our study offers several advantages. First, it is not limited to a targeted protein list. Second, the in-depth characterization of individual peptides, such as glycation as a nonenzymatic posttranslational modification, provides another dimension of insight into diabetic changes at the peptide level, well before protein levels are measurably changing.

**Figure 6:**
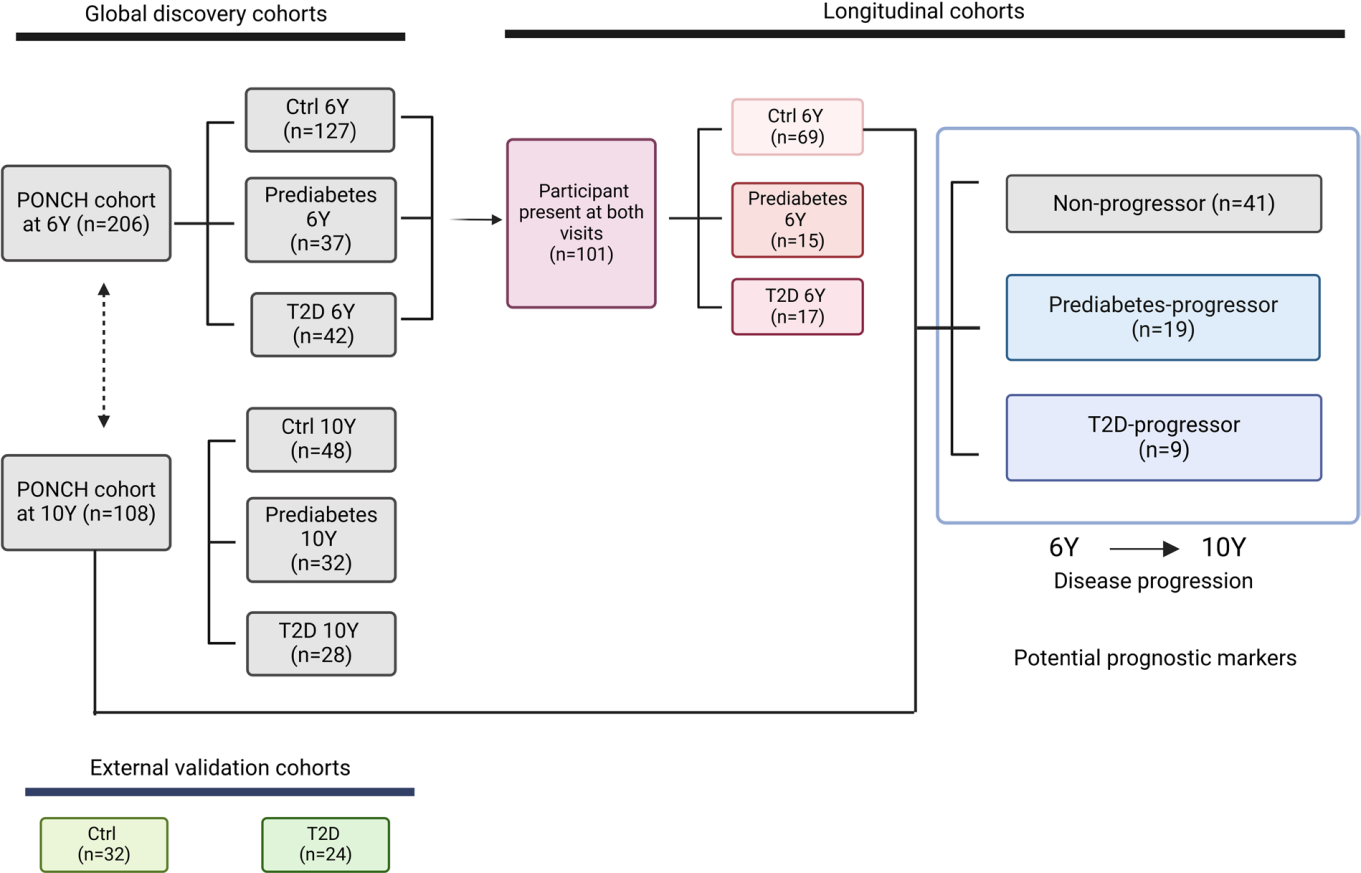
Summary of experimental cohort and corresponding analyses.

Highlighting the uniqueness of our study cohort, independent proteomics studies with a matching population of patients with a history of GDM were not available in the public domain. Therefore, we investigated our findings through multiple rigorous approaches to ensure data confidence and specificity (Figure 6). Using an ML method, we confirmed disease progression signatures by identifying important features. Additionally, our study cohort underwent subgroup splits based on nondemographic criteria, demonstrating the reproducibility of our results. We extended our analysis to an independent general T2D population with unknown history of GDM, and we evaluated 2 external T2D studies (40, 41), revealing consistent findings. While not all the findings in Table 3 were aligned with those independent studies, this could be attributed to post-GDM T2D specificity; different demographic characteristics, such as sex, age, and BMI; duration of diabetic status; or smaller subpopulation sizes. To assess each protein candidate’s generalizability, we conducted a review of literature and publicly available annotation data, as discussed below and summarized in Supplemental Table 1.

Our analysis indicated that PON3 was downregulated in participants with T2D and associated with BMI. ML analysis bolstered our finding from the proteomic profiling, and the low level of PON3 was corroborated in our validation cohort. A recent study demonstrated that liver PON3 expression was low in a rat model of T2D (56). Following liraglutide (glucagon-like peptide-1 analog known to reduce BMI and insulin resistance, Novo Nordisk) treatment and improvement of metabolic features, PON3 levels increased in this model (56). Additionally, PON3-KO mice were found to be susceptible to obesity (57). Our study highlights the association between PON3, BMI, and T2D in post-GDM women and suggests that the level of PON3 is reduced with diabetic disease progression together with high BMI, likely after onset of the disease.

Similarly, PLTP was another protein highlighted by our longitudinal analysis and ML analysis. In our study, we found a decline in PLTP with T2D progression and a reduced level of PLTP in the T2D population at 10 years after GDM. Conversely, some studies in the diabetes-relevant field showed an opposite trend in PLTP (58–61). In our study, PLTP exhibited weak correlations solely with HDL and HOMA-IR (at the 10-year visit), and we observed no associations with other clinical parameters or protein changes in contrast to other T2D signature proteins. This could be specific to the post-GDM population, as our study cohort is unique, rarely displaying common T2D confounding diseases. Since recent exome sequencing proposed obesity-independent therapeutic targets for diabetes (62), our study paves the way for integrated omics analysis as a means of specifying obesity-independent biomarkers.

Further findings include IL1RAP and SHBG, shedding light on their antidiabetic roles in a post-GDM population. IL1RAP is a coreceptor of the IL-1 family that activates NF-κB and mitogen-activated protein (MAP) kinase signaling pathways. The soluble form of IL1RAP was shown to have antiinflammatory properties in blood (63), and multiple polymorphisms of IL1RAP have been linked to the development of inflammatory-related disorders such as obesity (63). Studies targeting IL-1 therapies have shown a beneficial effect of anti–IL-1 therapy in T2D (64), but understanding of the direct effect of IL1RAP on diabetes is not clear. Our data demonstrate that IL1RAP’s antiinflammatory role extends to the post-GDM population. We also propose SHBG as a protein signature discriminating T2D over normoglycemia. SHBG is an FDA-approved biomarker (65), and its lower levels have been specifically linked to early-onset GDM and insulin resistance (66, 67). Although low circulating SHBG has been consistently identified as a marker for T2D in both sexes in smaller studies and metaanalyses (67, 68), previous studies on SHBG have mostly been conducted in postmenopausal women (69). Additionally, our results highlight that SHBG could serve as both a diagnostic and prognostic marker for a wider population of T2D, including the post-GDM T2D group.

Understanding diabetic outcomes and the corresponding proteome changes enabled us to elucidate whether a given protein marker is differentially expressed before or after the onset of the disease. Our study highlighted SHBG, IGFBP2, and IGFBP6 as potential prognostic marker candidates. IGFBP2, has been extensively studied for its antidiabetic effects and as a potential T2D biomarker (17) and demonstrates a strong association with risk of T2D (70), although its causal relationship remains uncertain (17, 71). Our findings highlight a decline in IGFBP2 levels preceding the onset of T2D. IGFBP6, also belonging to the IGFBP family, emerges as a prognostic marker candidate (17, 71). As opposed to IGFBP2, we observed a weak correlation between IGFBP6 and BMI, suggesting its potential utility as a marker for T2D development in women with other risk factors besides high BMI, such as compromised insulin production.

Furthermore, prediabetes-specific proteome features were demonstrated in proteins such as VCAM1 and severity-dependent common proteome with T2D, SHBG, or C3. A previous study in the Fenland cohort with both sexes participating has identified C3 and SHBG, as plasma IGT signatures (10). Downregulation of VCAM1 in prediabetes was surprising because VCAM1, an intercellular adhesion molecule, was previously reported to increase in diabetes due to its role in renal and other diabetic-driven complications (72). These features can be considered for future studies with larger cohorts to validate prognostic markers and early diagnostic markers for prediabetes, which is usually asymptomatic.

In summary, targeted screening and intervention within this post-GDM population are key, given the high risk of developing T2D, which is typically a silent disease for many years, causing late complications before diagnosis. Our study identifies protein signatures for the development of T2D and prediabetes that can facilitate appropriate management strategies to prevent the onset of diabetes or early intervention in post-GDM women.

### Limitations of the study.

We performed an unpowered exploratory omics analysis in a retrospective cohort. Our study only included women with a history of GDM and did not include women with no history of GDM, considered controls. The quantitation of proteins was relative quantitation, not absolute quantitation. However, these limitations are less concerning, as GDM versus non-GDM cohorts have been studied previously, and GDM biomarkers have been proposed, including CRP, PRG4, select apolipoproteins, and complement proteins (18, 19). One of the limitations of the study is the absence of proteomics datasets from the same patients before the diagnosis of GDM, due to practical challenges of identifying women at this stage. However, this study provides valuable insights by specifically focusing on the progression of diabetes during the postpartum-stage disease progression. Therefore, we believe that using post-GDM nonglycemic individuals as the control group is appropriate for our study objectives. Additionally, to ensure the reliability of our quantitation strategy using MS, we confirmed our MS-based results with a widely used biochemical measurement platform (ELISA) (Supplemental Figure 1).

## Methods

### Sex as a biological variable.

This study involved only female participants, specifically women who had a history of GDM. The focus on this population is due to their increased risk of T2D after pregnancy. The findings are primarily relevant to women, as GDM is a condition unique to that sex. While the biological processes driving the development of T2D may overlap between sexes, further studies are needed to confirm the relevance of these findings in men.

### Study participant details.

The PONCH cohort was composed of 237 women of mixed ethnicity in Sweden (50% of participants not of Nordic descent) with a history of GDM. Serum samples for proteomics were collected at the 6-year and 10-year follow-up visits. All participants provided informed consent. The study was conducted in accordance with the Declaration of Helsinki and was approved by the ethics review board of the University of Gothenburg (no. 402-08/750-15).

Clinical and biochemical characteristics of the participants were obtained as described previously (21). Biochemical analyses were performed at the Clinical Chemistry Laboratory, Sahlgrenska University Hospital. Body composition was measured with the Bod Pod Gold Standard system (Bod Pod 2007 A, Life Measurement) and software versions 4.2.1 and 5.2.0. For more details see Supplemental Methods. Participants were classified as healthy (normoglycemic), prediabetic, or T2D based on known diabetes in clinical interviews or newly diagnosed based on fasted, 2-hour glucose after a 75 g OGTT, and HbA1c in accordance with 1999 WHO guidelines (2). Supplemental Figure 1B shows the number of participants diagnosed by each criterion. The prediabetic classification included impaired fasting (fasting glucose 6.1–6.9 mmol/L) and IGT (2-hour glucose 7.8–11.0 mmol/L).

T2D and healthy control serum samples for validation were purchased from Proteogenex and obtained from AstraZeneca Research Specimen Collection Program (RSCP).

### Sample processing.

Serum was collected and frozen at −80°C before sample processing. At the time of processing, hemolyzed samples were excluded by visual inspection. Depletion was performed using 7 μL serum and 300 μL of Top14 Abundant Protein Depletion Resin (A36372, Thermo Fisher Scientific) per sample in a 96-well plate format. Samples were randomized based on each donor’s OGTT status so that healthy patients and those with prediabetes and diabetes were represented on each plate. When paired samples from a given individual were present, they were randomized to the same plate. In the validation cohort, 10 μL of serum samples were processed with 400 μL of Top14 Abundant Protein Depletion Resin. The depleted serum was processed based on the Easypep digestion protocol (A45733, Thermo Fisher Scientific). Quality control samples were prepared within a plate and across plates (Supplemental Figure 1).

### LC-MS/MS analysis and DIA Spectra analysis.

LC-MS/MS analyses were conducted on an Orbitrap Exploris480 mass spectrometer (Thermo Fisher Scientific) coupled with an UltiMate 3000 RSLCnano System, an EASY-Spray Source.

Library-free DIA data analysis was performed by Spectronaut (version 15 and version 18 for the study cohort and validation cohort, respectively). The default setting of DirectDIA analysis was performed mostly according to the standard workflow in Spectronaut (Biognosys). Detailed settings are described in the Supplemental Methods.

### Data preprocessing and imputation of missing data for ML analysis.

To perform the supervised ML analysis, 3 scenarios were considered; T2D-progressors, prediabetes-progressors, and prognostic marker candidates for T2D. We filtered out any clinical covariates that did not have values for all individuals of the minority class (i.e., cases) in each of the 3 scenarios. Covariates that are retained after this filtering step have, at most, 5% of values missing, in each scenario. The precise number of samples in each class in each scenario is provided in Supplemental Figure 5A. Missing data were imputed with the median value of the respective feature. More details around the method can be found in Supplemental Methods.

### Supervised learning models, fine-tuning and feature importance in ML analyses.

We began the ML analysis with a preliminary model selection step from which we observed random forest to be the highest performing (based on classification AUC), relative to logistic regression and gradient boosting (Supplemental Figure 5B). We selected to use a random forest model with 100 estimators to model the proteomic and clinical covariates, since it has comparable or better performance than the more complex models. After selecting which clinical covariates to include in the feature selection step, we explored the predictive AUC across the 3 scenarios (Supplemental Figure 5, Figure 3D, and Figure 4G).

To estimate the final measure of feature importance, we averaged across 200 random forests by taking the median feature importance value over each of these iterations as the final value of feature importance for a given feature. Such an approach mitigates against the inherent stochasticity of the learning algorithm for random forests, while also maintaining parallelizability. We then generated a normalized form of feature importance (varying between 0 and 1) through exploiting a null distribution of feature importance generated by permuting the labels over all samples, so that the probability of a single data point retaining its class label diminished as the number of samples increased.

### Statistics.

We performed exploratory proteomics analysis in a retrospective cohort study. No methods were used to predetermine sample size. The analysts were not blinded to the clinical data. Perseus version 1.6.15.0 was used for the downstream analyses. The data were log_2_ transformed, and differential abundance analysis was performed with Welch’s 2-tailed *t* test with s0 set to 0 in Perseus. Proteins with Benjamini-Hochberg FDR < 0.1 were considered significant. The rationale for relaxed FDR cut-off was that we were working with unique participant material and considered the omics analysis as hypothesis generating. Proteins with *P* < 0.05 were also reported when they were supported by multiple lines of evidence (different subgroup analyses). Analysis results were reproduced by an independent validation programmer in R v4.0.2. For multiple group comparisons, an initial 1-way ANOVA with Benjamini-Hochberg FDR for truncation was performed using Perseus (Supplemental Data File 4). For pairwise comparisons, adjusted *P* values were calculated using Brown-Forsythe and Welch ANOVA tests (1-way) with Dunnett’s T3 multiple comparisons and visualized in Figure 2 using GraphPad Prism v9. Wilcoxon signed-rank test was conducted to compare the changes in BMI and HOMA-IR within participants. Sensitivity analysis was performed with linear regression adjusted for age and BMI. Sensitivity analysis was performed using 2 linear regression models for T2D-associated proteins using R.4.2.0. The first model was adjusted for age and BMI, and the second model additionally accounted for metformin and insulin treatment. For longitudinal progression change in diabetic progression, a sensitivity analysis was conducted to adjust for BMI. GraphPad Prism v9 and ggplot2 (ISBN 978-3-319-24277-4) were used to make plots. The Tukey method was used to generate box-and-whisker plots via GraphPad Prism. IPA (QIAGEN) was used for the enrichment analysis of differentially expressed proteins (Fisher’s exact test corrected for background frequencies of IPA pathway terms among all quantified serum proteins).

In the validation cohort, differences between individuals with T2D and healthy participants were assessed with linear regression adjusted for batch by serum collection year and hemolysis.

Differences in response to OGTT among subgroups of healthy participants at 6 years were evaluated with a mixed-effect linear model. The model included time after OGTT, patient subgroup, and interaction between time and patient subgroup as fixed effects, with factor encoding and participant as random intercepts. Model quality control was based on residual diagnostics and correlation between measured and predicted values. AUCs were calculated with trapezoid approximation. We used R packages lme4 v 1.1-23, lmerTest v 3.1-2, and DescTools 0.99.49.

### Study approval.

The PONCH study was conducted in accordance with the Declaration of Helsinki and was approved by the ethics review board of the University of Gothenburg (402-08/750-15). All participants provided informed consent. For the validation cohort, samples were purchased from Proteogenex and obtained from AstraZeneca RSCP. Informed consent was obtained from each individual at enrollment.

### Data availability.

MS proteomics data are available at the MassIVE repository, a ProteomeXchange Consortium partner, with the dataset identifier MSV000092252. The raw data used to make the graphs found in the manuscript’s figures are available in the accompanying Supporting Data Values file.

## Author contributions

HSC designed the proteomics experiments; performed, analyzed, interpreted the data; and wrote the manuscript. LM and DV performed ML analyses, generated data and figures, and wrote the corresponding method texts. MG performed statistical analyses and generated data. AH, DB, UAH, and KW set up the research collaboration and designed this project. AH and UAH were responsible for the clinical study. VAH helped design and perform proteomics acquisitions, analyzed data, and generated corresponding figures. VAH, KW, UAH, SH, and RBV interpreted the results and reviewed and edited the manuscript. SH, KW, UAH, DB, BGC, and AH supervised the project and reviewed the manuscript.

## Supplementary Material

Supplemental data

Supplemental tables 1-8

Supporting data values

## Figures and Tables

**Table 1:**
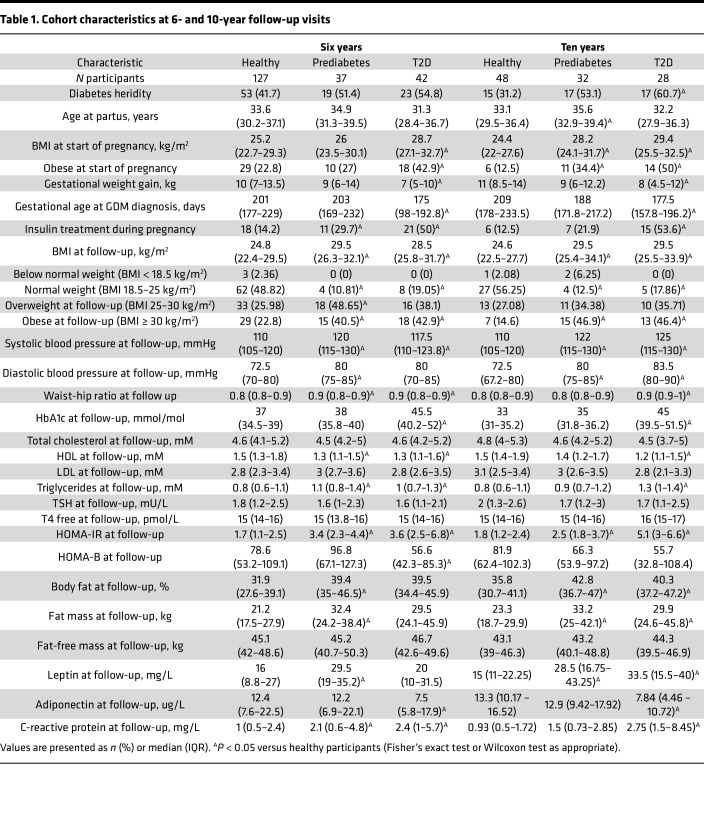
Cohort characteristics at 6- and 10-year follow-up visits

**Table 2:**
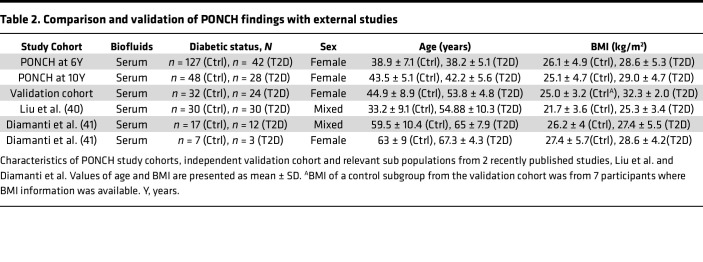
Comparison and validation of PONCH findings with external studies

**Table 3:**
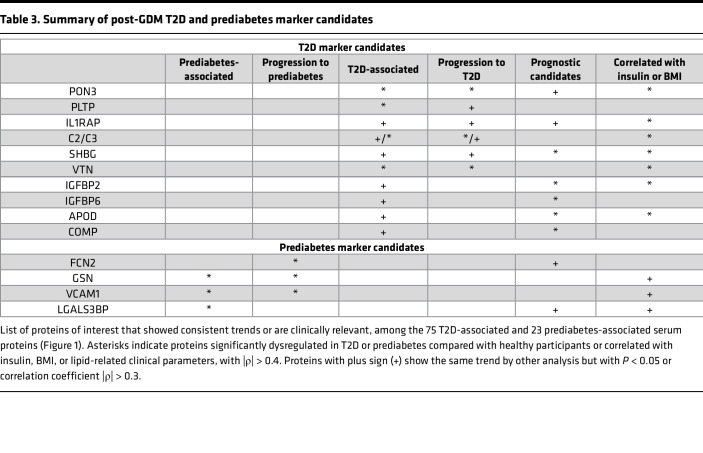
Summary of post-GDM T2D and prediabetes marker candidates

## Version 1 11/26/2024

In-Press Preview

## Version 2 01/23/2025

Electronic publication

## Version 3 01/27/2025

After publication of the manuscript, the authors notified the journal of an error in Figure 6. A previous version of the paper referenced an internal cohort that was subsequently removed from the final version of the manuscript. Figure 6 has been updated to more accurately reflect the study design
