# Longitudinal clinical and proteomic diabetes signatures in women with a history of gestational diabetes

**DOI:** 10.1172/jci.insight.191787

**Published:** 2025-02-10

**Authors:** Heaseung Sophia Chung, Lawrence Middleton, Manik Garg, Ventzislava A. Hristova, Rick B. Vega, David Baker, Benjamin G. Challis, Dimitrios Vitsios, Sonja Hess, Kristina Wallenius, Agneta Holmäng, Ulrika Andersson-Hall

Original citation *JCI Insight*. 2025;10(2):e183213. https://doi.org/10.1172/jci.insight.183213

Citation for this corrigendum: *JCI Insight*. 2025;10(3):e191787. https://doi.org/10.1172/jci.insight.191787

After publication of the manuscript, the authors notified the journal of an error in Figure 6. A previous version of the paper referenced an internal cohort that was subsequently removed from the final version of the manuscript. Figure 6 has been updated to more accurately reflect the study design. The HTML and PDF files have been updated. 

The authors regret the error.

## Version 1 02/10/2025

Electronic publication

