# Supplementary material for: Longitudinal clinical and proteomic diabetes signatures in women with a history of gestational diabetes
Source: JCI Insight. 2024 Nov 26;10(2):e183213. doi: 10.1172/jci.insight.183213 (PMC11790031; doi:10.1172/jci.insight.183213)
Supplement: Supplemental data [file jciinsight-10-183213-s239.pdf]

Supplemental Information

Longitudinal clinical and proteomic diabetes signatures in women with a history of gestational diabetes

Heaseung Sophia Chung[1,*], Lawrence Middleton[2], Manik Garg[2], Ventzislava A. Hristova[1§], Rick B. Vega[3], David Baker[4], Benjamin G. Challis[5], Dimitrios Vitsios[2], Sonja Hess[1], Kristina Wallenius[6], Agneta Holmäng[7], Ulrika Andersson-Hall[7,*]

[1]Dynamic Omics, Centre for Genomics Research, Discovery Sciences, BioPharmaceuticals R&D, AstraZeneca, Gaithersburg, MD, USA

[2]Centre for Genomics Research, Discovery Sciences, BioPharmaceuticals R&D, AstraZeneca, Cambridge, UK

[3]Early Clinical Development, Early CVRM, BioPharmaceuticals R&D, AstraZeneca, Gaithersburg, MD, USA

[4]Bioscience Metabolism, Early CVRM, BioPharmaceuticals R&D, AstraZeneca, Cambridge, UK

[5]Translational Science and Experimental Medicine, Early CVRM, BioPharmaceuticals R&D, AstraZeneca, Cambridge, UK

[6]Bioscience Metabolism, Early CVRM, BioPharmaceuticals R&D, AstraZeneca, Gothenburg, Sweden

[7]Institute of Neuroscience and Physiology, Department of Physiology, Sahlgrenska Academy, University of Gothenburg, Gothenburg, Sweden

[§]Present affiliation: Precision Medicine, Amgen, Rockville, MD, USA

*Correspondence: H. Sophia Chung: 1 Medimmune Way, Gaithersburg, MD 20878, USA, +1-301-398-2433, sophia.chung@astrazeneca.com; Ulrika Andersson-Hall: Institute of Neuroscience and Physiology, Box 432, Sahlgrenska Academy, University of Gothenburg, 405 30 Gothenburg, Sweden. +46-730 315 918, ulrika.andersson.hall@gu.se

Table of Content:

- **Extended Methods**
- **Key Resources Table**
- **Supplementary Table 1**
- **Supplementary Figures**
    - Supplementary Figure 1. Assessment of sample quality for proteomics analysis and protein quantification precision
    - Supplementary Figure 2. Cross-sectional comparison of serum proteome of T2D and prediabetic vs. healthy post-GDM women in multivariate regression analysis adjusted for age and BMI
    - Supplementary Figure 3. Expanded sets of proteins associated with diabetes severity and clinical traits
    - Supplementary Figure 4. Progression to diabetes between 6-year and 10-year follow-up visits was associated with increase in BMI and HOMA-IR but not markers of end organ damage
    - Supplementary Figure 5. Machine Learning (ML) analysis
    - Supplementary Figure 6. Differential expression of 6 protein markers
    - Supplementary Figure 7. Differential expression of non-progressor *vs.* progressors at 6-year visit, when their glycemic status was normal
    - Supplementary Figure 8. Proteomic profiling of a validation cohort and replication of the findings from PONCH study in two independent analyses

Extended Methods

Drop-out analysis: women included at 6 years only vs. those included at both visits

Drop-out analysis was performed by comparing 6-year data for women who attended only the 6-year visit with women who attended both the 6-year and 10-year visits. Statistical tests used were Students t-test for continuous variables and Chi2 test for glucose tolerance group. The drop-out analysis indicated no selection bias between women included in the 6-year analysis only and those included in both the 6- and 10-year analyses. There were no significant differences in BMI (27.5 kg/m2 vs 27.0 kg/m2, p=0.50), fasting glucose (6.2 mM vs 6.0 mM, p=0.47), 2-hour glucose (6.6 mM vs 6.3 mM, p=0.45), HbA1c (41mmol/mol vs 40mmol/mol, p=0.35) or glucose tolerance group (p=0.67) at the 6-year visit between women included at 6 years only and those included at both visits, respectively.

Clinical and biochemical measurements

Glucose, insulin, cholesterol, T4, TSH, HDL, LDL, hs-CRP, leptin and adiponectin were all analysed at the Clinical Chemistry Laboratory, Sahlgrenska University Hospital (accredited in accordance with the International Standard ISO 15189:2007). Leptin (Human Leptin Quantikine, R&D Systems, Minneapolis, MN; interassay coefficient of variation, 8.0% at 9 mg/l) and adiponectin (Human Adiponectin ELISA kit, Millipore, Billerica, MA; interassay coefficient of variation, 7.0% at 10.5 mg/l) were analysed using ELISA, the rest with a Cobas Modular system (Roche Diagnostics, Risch, Switzerland). HOMA-IR was calculated as (fasting glucose × fasting insulin)/22.5 and HOMA-B as (20 × fasting insulin)/(fasting glucose − 3.5). Body composition was measured with the Bod Pod Gold Standard system (Bod Pod 2007 A, Life Measurement, Concord, CA) and software versions 4.2.1 and 5.2.0. The coefficient of variation (CV) for body composition measurements on our equipment was 2.4%.

LC-MS/MS analysis for global proteomics

LC-MS/MS analyses were conducted on an Orbitrap Exploris480 mass spectrometer (Thermo Fisher Scientific) coupled with an UltiMate 3000 RSLCnano System, an EASY-Spray Source. Sample injections were loaded onto a 0.075 mm × 20 mm Acclaim™ PepMap™ 100 C18 trap column (Thermo Fisher Scientific) and a 0.15 mm × 150 mm ES906 EASY-Spray analytical

column (Thermo Fisher Scientific). The peptides were eluted at a flow rate of 350 nl/min over a 30-min gradient, from 4 to 24% solvent B (15 min), 24 to 40% solvent B (5.5min), 40 to 98% solvent B (4.5 min), and 98 to 2% solvent B (5 min). Solvent A was composed of 0.1% formic acid (FA) in water, and solvent B was composed of 0.1% FA in 100% acetonitrile.

The full MS survey scan [m/z (mass/charge ratio) 350 to 1200] was acquired on an Orbitrap analyzer at a resolution of 120,000 and with a normalized automatic gain control (AGC) target of 100%. The maximum injection time for MS scans was 45 ms. Scans for DIA were performed with the higher energy collisional dissociation (HCD) collision energy set to 30% with an isolation window of 21 m/z and window overlap of 1 m/z. Other parameters include resolution of 30,000, number of scans of 40, a normalized AGC target of 2000% and a maximum injection time of 100 ms.

DIA Spectra analysis

Library-free DIA data analysis was performed by Spectronaut (version 15). The default setting of directDIA analysis was performed mostly according to the standard workflow in Spectronaut (Biognosys). In brief, carbamidomethylation of cysteine was set as a fixed modification and the variable modification setting included oxidation of methionine and acetylation at N-terminus. The MS mass tolerance was set as dynamic (correction factor for window 1). The False Discovery Rate (FDR) was set to 1% at the peptide precursor level. Quantification was by MS2 and major and minor group quantities were set to mean peptide and mean precursor quantity. Precursor filtering was based on q-percentile of 0.2 and missing values were imputed based on a random value derived from the distribution of quantities for each analysis individually. Cross-run normalization was performed based on a retention time-dependent local regression model. Analysis of the validation cohort was performed with the default setting of directDIA by Spectronaut (version 18). Subsequently, 428 protein groups (avg. of 26 precursors per protein groups) and 859 protein groups (avg. of 22 precursors per protein groups) were identified from the PONCH study cohort, and the validation cohort, respectively. Protein intensities were log2 transformed, averaged per technical triplicate for each participant and quantile normalized. Proteins with missing intensities in > 15 % of the participants were excluded from analysis. The remaining missing intensities were imputed with sequential imputation method (R rrcovNA::impSeq).

LC-MS/MS analysis of glycated peptides

LC-MS/MS analyses were conducted on an Orbitrap Exploris480 mass spectrometer (Thermo Fisher Scientific) coupled with an UltiMate 3000 RSLCnano System, an EASY-Spray Source. Sample injections were loaded onto a 0.075 mm × 20 mm Acclaim™ PepMap™ 100 C18 trap column (Thermo Fisher Scientific) and a 0.15 mm × 150 mm ES906 EASY-Spray analytical column (Thermo Fisher Scientific). Peptides were eluted at a flow rate of 350 nl/min over a 30-min gradient. The full MS survey scan [m/z (mass/charge ratio) 400 to 1800] was acquired on an Orbitrap analyzer at a resolution of 60,000 and with a normalized automatic gain control (AGC) target of 300%. The maximum injection time for MS scans was 100 ms. MS2 Scans for data dependent acquisition (DDA) were performed with the higher energy collisional dissociation (HCD) collision energy set to 35% with an isolation window of 1.6 m/z. Other parameters for MS2 scan include resolution of 15,000, number of scans of 20, a normalized AGC target of 100% and a maximum injection time of 50 ms.

Data analysis of glycated peptides

The analysis was conducted using Spectronaut (version 19) as a library-based search with DIA .raw files obtained from the main proteomics analysis. The library was generated using two of the PONCH study DDA .raw files and integrated with in-house search histories. The settings for the spectral library generation followed the default settings pre-set in Spectronaut (version 19), with the exception of the following modification: fixed carbamidomethylation of cysteine and dynamic oxidation of methionine; and dynamic Amadori compound modification at lysine (162.05 Da). Quantification was achieved at peptide level and only peptides with missing values of less than 0.3 in each diabetic status were considered for downstream statistical analysis. Cross-sectional analysis was independently performed between T2D and healthy cohorts at 6 years and 10 years.

Patient definitions for Machine Learning (ML) analysis

We have adopted a binary classification framework, with class labels distinguishing between patients depending on which of the three scenarios is being considered. The first scenario (healthy-to-diabetic) was to find T2D-progressors-specific features and a positive label was assigned to T2D-progressors (those samples that were healthy at 6y and T2D at 10y) and a

negative label to non-progressors (those that stayed healthy) throughout the whole period. The features for the first scenario included the change in protein levels from 6y to 10y. The second scenario (healthy-to-prediabetic) was to identify prediabetes-progressor-specific features and a positive label was assigned to those samples that were healthy at 6y but developed to pre-diabetic at 10y and used the same data as the first scenario (i.e.; healthy-to-diabetic). The third scenario (termed as prognostic model) was to identify prognostic markers and the same labels were used as in the first scenario (i.e.; healthy-to-diabetic), however, using data only up until the 6y time point.

Data pre-processing and imputation of missing data

We excluded from the feature set any features associated with glucose, insulin or diabetes, to avoid circularity in our predictions from using any direct proxies of the prediction outcome (i.e. progression to diabetes/prediabetes). This resulted in a model that includes 49 clinical covariates (including BMI, waist-hip-ratio and age at partus), and 477 features in total (including 428 proteins). With regards to missing data, we were primarily concerned with missingness in samples in the minority class (i.e. T2D-progressors or prediabetes-progressors depending on the scenario). As such, we filtered out clinical covariates which do not obtain the minimum over minority classes for the three scenarios, i.e. a minimum of 9 samples in each class. Covariates that are retained after this filter had at most 5% of values missing, in each scenario. Precise number of samples in each class in each scenario is provided in Supplementary Figure 5A. Missing data was imputed with the median value across all values for that feature.

Supervised learning models and fine-tuning

The machine learning analysis started with a preliminary model selection step from which random forest performed best (based on classification AUC), relative to logistic regression and gradient boosting (Supplementary Figure 5B) During this preliminary phase only proteomic data was included (and no clinical covariates). To ensure robustness in the analysis, the AUC was estimated using stratified *k*-fold cross-validation (setting *k*=2 due to limited sample size) and repeating the AUC estimation over multiple within-class random shufflings. Such an approach ensures that different data values typically fall in different folds, while preserving the labelling of the data (as well as the class distribution between folds).

For each model, the three different parameter sets, denoted as 'Param set' 1, 2 or 3, explored were as follows:

1. Logistic regression regularisation parameter, varied in [1, 10, 100]

2. Gradient boosting number of trees, varied in [10,000, 1,000, 100]

3. Random forest number of trees, varied in [10,000, 1,000, 100]

The number of *k* -fold random shufflings performed varied depending on the computational cost of obtaining a single estimate of the AUC. For logistic regression (lower computational cost) this was set to n=100, though for gradient boosting and random forest (that increase in computational complexity with the number of trees) the number of shufflings n was defined so that its product with the number of trees was equal to 100,000 (so n=10 for 10,000 trees, n=100 for 1,000 trees etc.). We selected to use a random forest model with 100 estimators to model the proteomic and clinical covariates, since it had comparable or better performance than the more complex models (i.e. number of estimators=1,000 or 10,000) and had lower computational cost. Thus, the number of random shufflings in that case was 1,000.

After selecting which clinical covariates to include in the feature selection step, we explored the predictive AUC across the three scenarios (i.e. healthy-to-diabetic, healthy-to-prediabetic and prognostic). As per the model selection phase, multiple AUCs were obtained through repeated within-class random shufflings (set to 5,000 shufflings for these purposes).

Validating ML models on UK Biobank (UKB) data

All machine learning models were validated on a relevant, independent cohort identified using UK Biobank data (1). While the exact definition of the cohort inevitably differs from the cohort used in this study, we are able to derive a meaningful proxy to the cohort used in the PONCH study, i.e. by focusing on individuals that first had T2D (E11 in the ICD-10 nomenclature) at some point after GDM (O24.4). In particular, the cohort is isolated as follows: i) Identify individuals that had either T2D or GDM, then discard those that had T2D prior to GDM (47,232 individuals) ii) Identify those individuals that had GDM (O24.4) some of which may have no T2D diagnosis and some that went on to develop this (219 individuals left) iii) Subset those by those that have OLINK proteomic data available (26 individuals in total) (2). Based on this, perform a two-sided Mann-Whitney-U test of proteomic levels in the two groups – those that did not have a

subsequent T2D diagnosis and those that did, resulting in a p-value for 2,940 unique proteins. Of these we match 224 to proteins in the PONCH study.

Review of publicly available annotation data

We conducted a comprehensive review of publicly available genome/phenome-wide data derived from the UK Biobank, and publicly available gene annotation data, utilizing information from PheWAS (3), Mantis-ML (4), and MILTON (5). PheWAS and Mantis-ML both provide gene-level associations while MILTON aims to estimate biomarker signatures from protein data among other covariates. For PheWAS, we defined the phenotype ICD10 codes (specifically E11 Non-insulin-dependent diabetes mellitus and its subcategories) and considered a top 5% rank as indicative of substantial support for the gene/protein-association. Across the different resources, our analysis identified IL1RAP (p=0.0001515, % Rank: top 1%) as providing support from PheWAS, and VCAM1 (top 4%), VTN (top 4%), and IGFBP2 (top 2.5%) from Mantis-ML. Additionally, MILTON highlighted PON3 (top 0.2%) and APOD (top 0.6%).

Visualization

Figure 1A, Figure 3A and B, Figure 4A and D and Figure 6 were created with BioRender.com

Key Resources Table

REAGENT or RESOURCE	SOURCE	IDENTIFIER
Human serum fluid	PONCH study, the University of Gothenburg	402-08/750-15
Antibodies		
Top14 abundant protein depletion resin	Thermo Scientific	A36372
Critical commercial assays		
Leptin Quantikine	R&D Systems	DLP00
Human Adiponectin ELISA kit	Millipore	EZHADP-61K
EasyPep™ MS Sample Prep Kits	Thermo Scientific	A45733
Deposited data		
Raw mass spectrometry data	This study	MSV000092252
Software and algorithms		
Bod Pod Gold Standard system software (versions 4.2.1 and 5.2.0)	Life Measurement Inc.	
Spectronaut (version 15, 18 and 19)	Biognosys AG	https://biognosys.com/software/spectronaut/
Perseus (version 1.6.15.0)	Tyanova et al., 2016(6)	https://maxquant.net/perseus/
R & RStudio	R Foundation	https://www.r-project.org/
GraphPad Prism 9	GraphPad	https://www.graphpad.com/
Ingenuity Pathway Analysis	QIAGEN	https://www.qiagenbioinformatics.com/products/ingenuitypathway-analysis

Supplementary Table 1. Summary of post-GDM T2D and prediabetes marker candidates (Extended version of Table 3). List of proteins of interest that showed consistent trends or are clinically relevant, among the 75 T2D-associated and 23 prediabetes-associated serum proteins (Figure 1). Asterisk (*) was displayed for proteins significantly dysregulated in T2D or prediabetes compared to healthy participants or correlated with insulin, BMI or lipid-related clinical parameters, with |ρ| >0.4, proteins with plus sign (+) shows the same trend by other analysis but with raw p<0.05 or correlation coefficient |ρ| >0.3.

T2D marker candidates							
	PONCH study				Reference		
	**T2D-associated**	**Progression to T2D**	**Prognostic candidates**	**Correlated with insulin or BMI**	**T2D-association identified**	**GDM-association identified**	**Genome/Phenome-wide T2D-association**
PON3	*	*	+	*	(7, 8)	(9)	(5)
PLTP	*	+			(10-13)	(14)	
IL1RAP	+	+	+	*			(3)
C2/C3	+/*	*/+		*	C2(15)<br>C3(16)		
SHBG	+	+	*	*	(17-21)	(17, 22, 23)	
VTN	*	*		*	(16, 24)	(14, 25)	(4)
IGFBP2	+		*	*	(7, 8, 26-28)	(9, 29)	(4)
IGFBP6	+		*				
APOD	+		*	*			(5)
COMP	+		*				
**Prediabetes marker candidates**							
	**PONCH study**				**Reference**		
	**Prediabetes-associated**	**Progression to prediabetes**	**Prognostic candidates**	**Correlated with insulin or BMI**	**Prediabetes or T2D-association**	**GDM-association identified**	**Genome/Phenome-wide T2D-association**
FCN2		*	+				
GSN	*	*		+	(21)	(23)	
VCAM1	*	*		+	(30)	(31)	(4)
LGALS3BP	*		+	+	(32)		

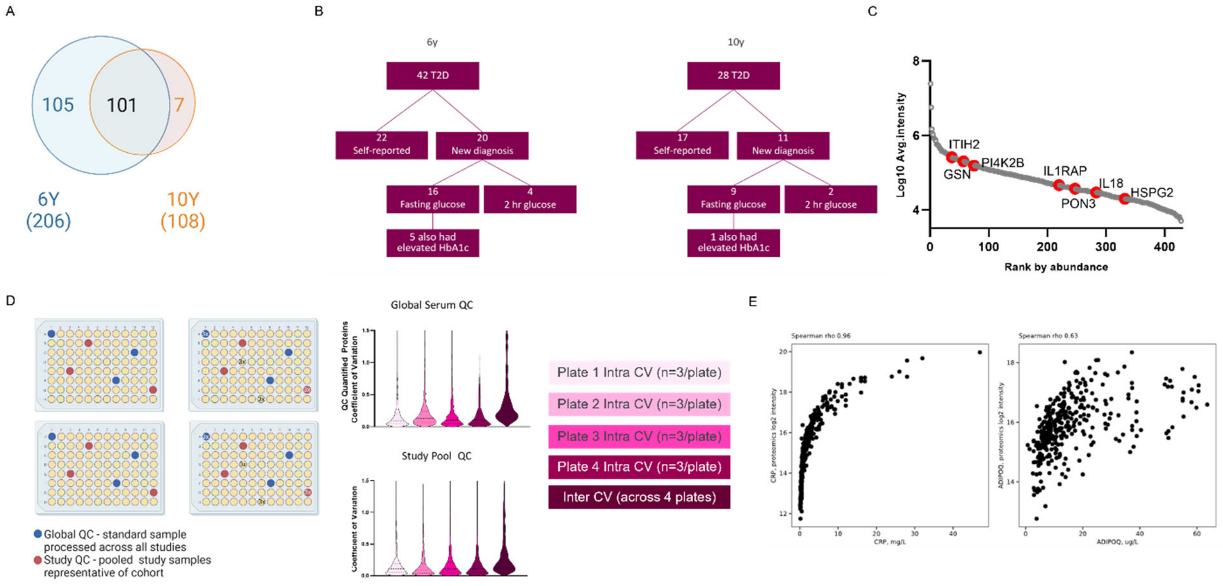

Supplementary Figure 1. Assessment of sample quality for proteomics analysis and protein quantification precision

(A) The cohort for proteomics analyses consists of 213 participants who contributed samples at least once at a follow-up visit. Samples from 7 participants collected at the 10-year follow up visit were found to be hemolyzed and excluded from analyses.

(B) Breakdown of the number of participants diagnosed to have T2D by each criterion

(C) LC-MS/MS DIA global proteomics identified and quantified more than 400 protein groups whose intensities spanned over four orders of magnitude. Protein examples discussed in this study are labeled in red.

(D) Triplicates of global and study-specific pooled serum were processed with each sample plate/batch (left) and revealed a high technical reproducibility (right) based on low intra- and inter-plate coefficient of variation (CV).

(E) Tight correlation between protein quantity measured by our LC-MS/MS workflow and ELISA diagnostic test measurements confirms robustness of our method. Spearman rank correlation coefficient ρ = 0.96, (C-reactive protein (CRP), left), and ρ = 0.63 (adiponectin (ADIPOQ), right).

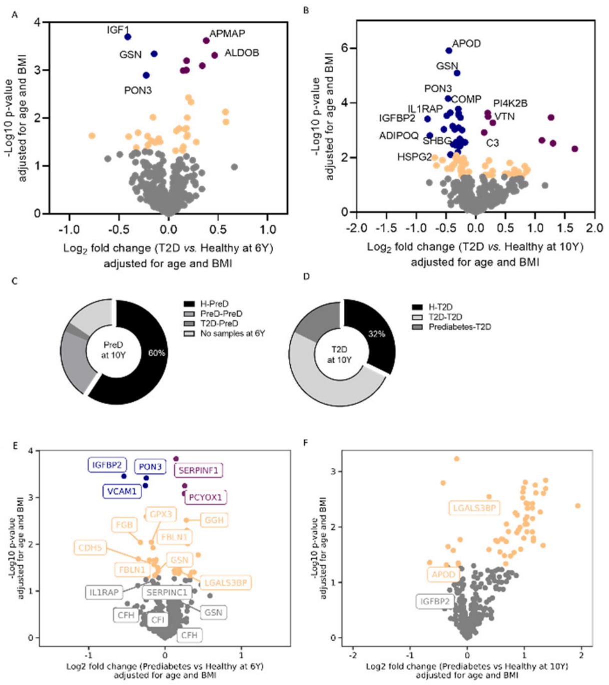

Supplementary Figure 2. Cross-sectional comparison of serum proteome of T2D and prediabetic vs. healthy post-GDM women in multivariate regression analysis adjusted for age and BMI

(A-B) Volcano plots of age and BMI adjusted analysis comparing the serum proteomes of T2D *vs.* healthy participants at 6-year (A) and 10-year (B) follow-up visits. The log2 fold change in protein abundance is displayed on the x axis and the –log10 p-value from multivariate linear regression adjusted for age and BMI on the y axis. Color code is based on raw $p < 0.05$ (in yellow), and decreased levels compared to healthy women (in blue) and increased levels compared to healthy women (in purple) significantly. Some proteins could be represented by more than one protein group (spectra matched different isoforms e.g., CFH) and therefore occur in the plot twice.

(C-D) Historic diabetic status of prediabetic (n=32, C) and T2D populations (n=28, D) at 10-year visit. Sixty percent of prediabetic participants at 10 years (n=27, where samples for the proteomics analysis were available for both visits) were healthy at 6 year and less than half of them stayed in the prediabetic status at both time points. However, only 30% of T2D participants at 10-year visit were healthy at 6-year visit.

(E-F) Volcano plots in age and BMI adjusted analysis comparing the serum proteomes of prediabetes *vs.* healthy participants at 6-year (E) and 10-year (F) follow-up post-GDM visits, displayed in the same way as in A-B.

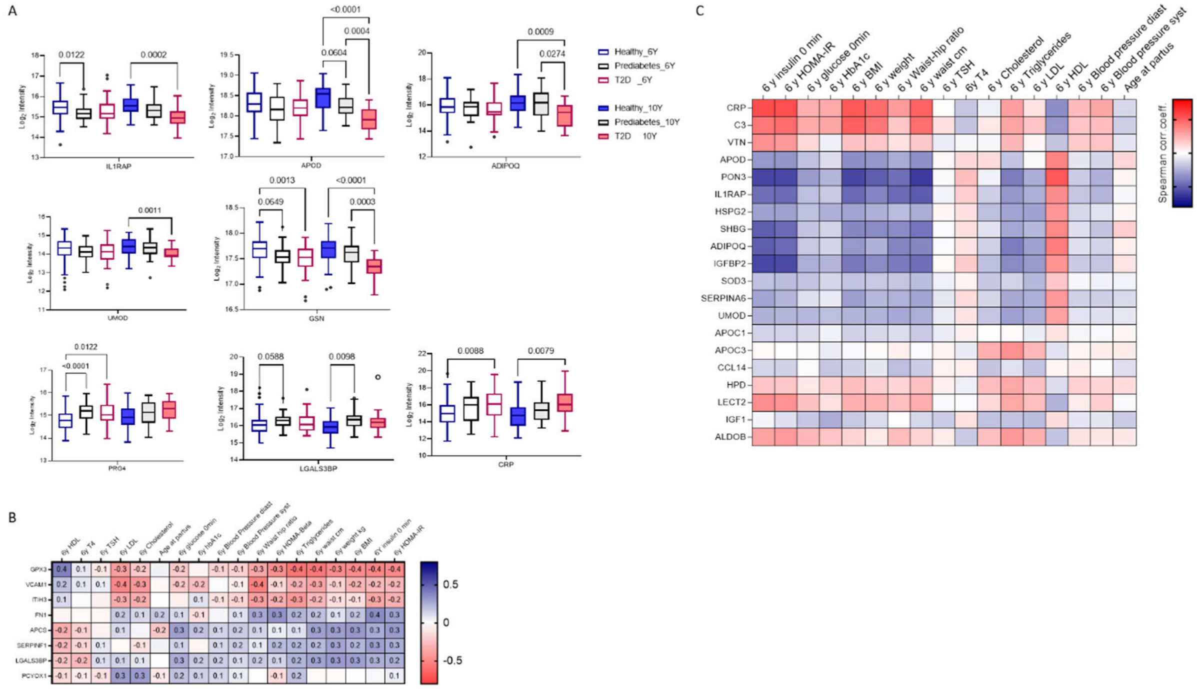

Supplementary Figure 3. Expanded sets of proteins associated with diabetes severity and clinical traits.

(A) Intensities of the selected proteins across subpopulations (ANOVA test, adj. p-value <0.05 at either visit). While IL1RAP, gelsolin (GSN), apolipoprotein D (APOD), ADIPOQ and uromodulin (UMOD) displayed a trend decreasing with diabetic status, PRG4 and CRP increased. Log2 intensity of participants in each subpopulation was displayed as a box and whisker plot showing median and IQR. Galectin-3-binding protein (LGALS3BP) was a prediabetic-specific protein in Fig 1. Adjusted p-value by Brown-Forsythe and Welch ANOVA tests and Dunnett's T3 multiple comparisons test were labeled if <0.1.

(B) Correlation heatmap of the selected proteins picked up in the prediabetic proteome analyses (Figure 1G), and clinical parameters measured at 6-year post GDM. Spearman's rank correlation was used to calculate the coefficient ρ and row clustering was based on log2 intensity of the protein.

(C) Expanded correlation heatmap of selected post-GDM T2D markers from Figure 1B-C with clinical characteristics of all participants at 6 years follow-up visits. Coefficient ρ and row clustering were calculated in the same way described in (B)

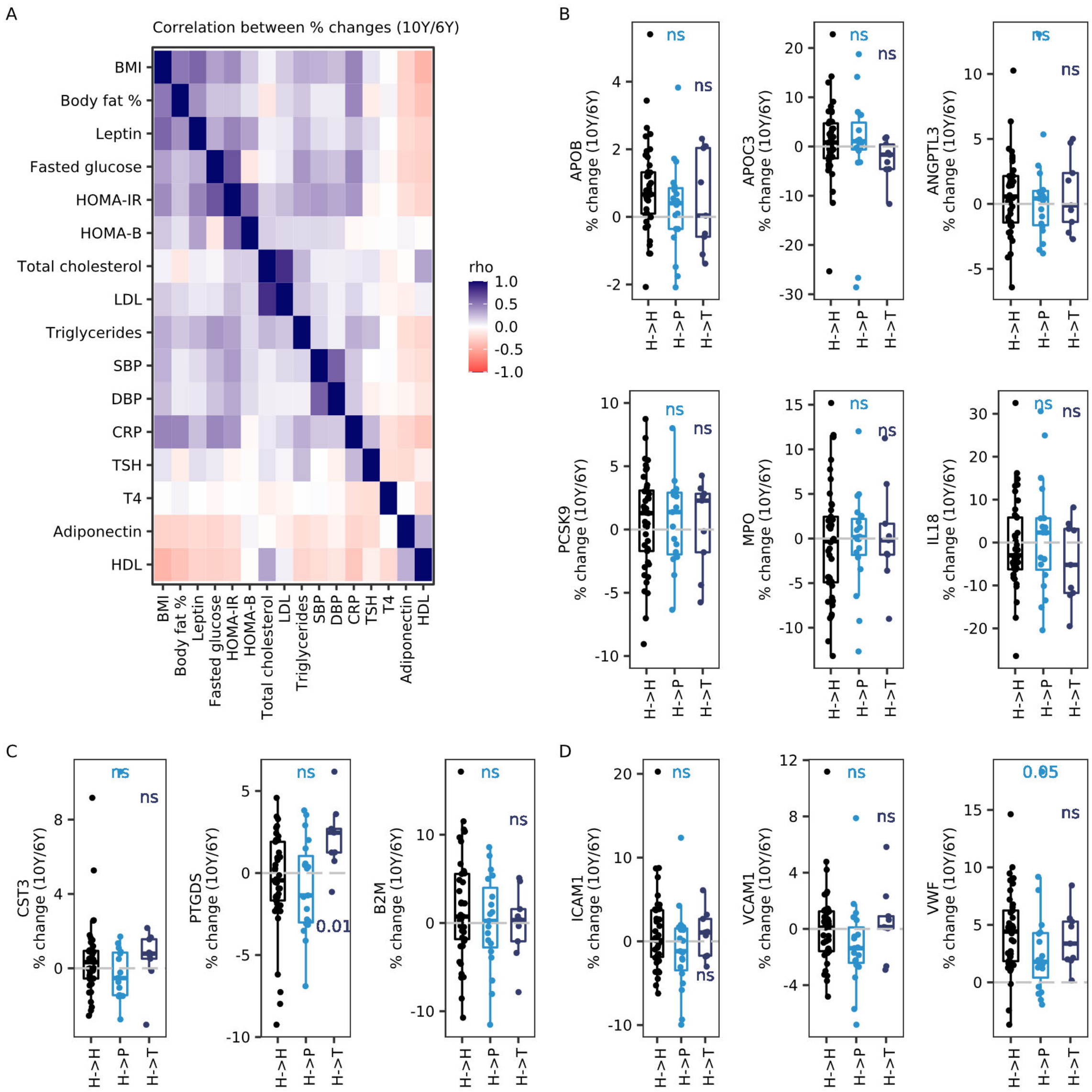

Supplementary Figure 4. Progression to diabetes between 6-year and 10-year follow-up visits was associated with increase in BMI and HOMA-IR but not markers of end organ damage.

(A) Changes in other clinical parameters correlated with changes in BMI and HOMA-IR.

(B-D) Percent change in biomarkers of cardiovascular risk and kidney and endothelial dysfunction did not increase in participants who progressed towards diabetes (sub-cohort #2: “H->P” and sub-cohort #3: “H->T”) between 6- and 10-year visits, compared to non-progressors (sub-cohort #1: “H->H”). The plots show within-individual changes calculated from serum proteins reflecting cardiovascular risk (B) kidney filtration function (C), and endothelial dysfunction (D). Numbers indicate nominal p-values in Wilcoxon test without adjustment for multiple testing, “ns” stands for p-value >= 0.05. Participants who were healthy at 6- and 10-

years (sub-cohort #1, n=41); healthy at 6 years but pre-diabetic at 10 years (sub-cohort #2, n=19); healthy at 6 years but T2D at 10 years (sub-cohort #3, n=9) and contributed paired proteomics samples at both 6 and 10 years.

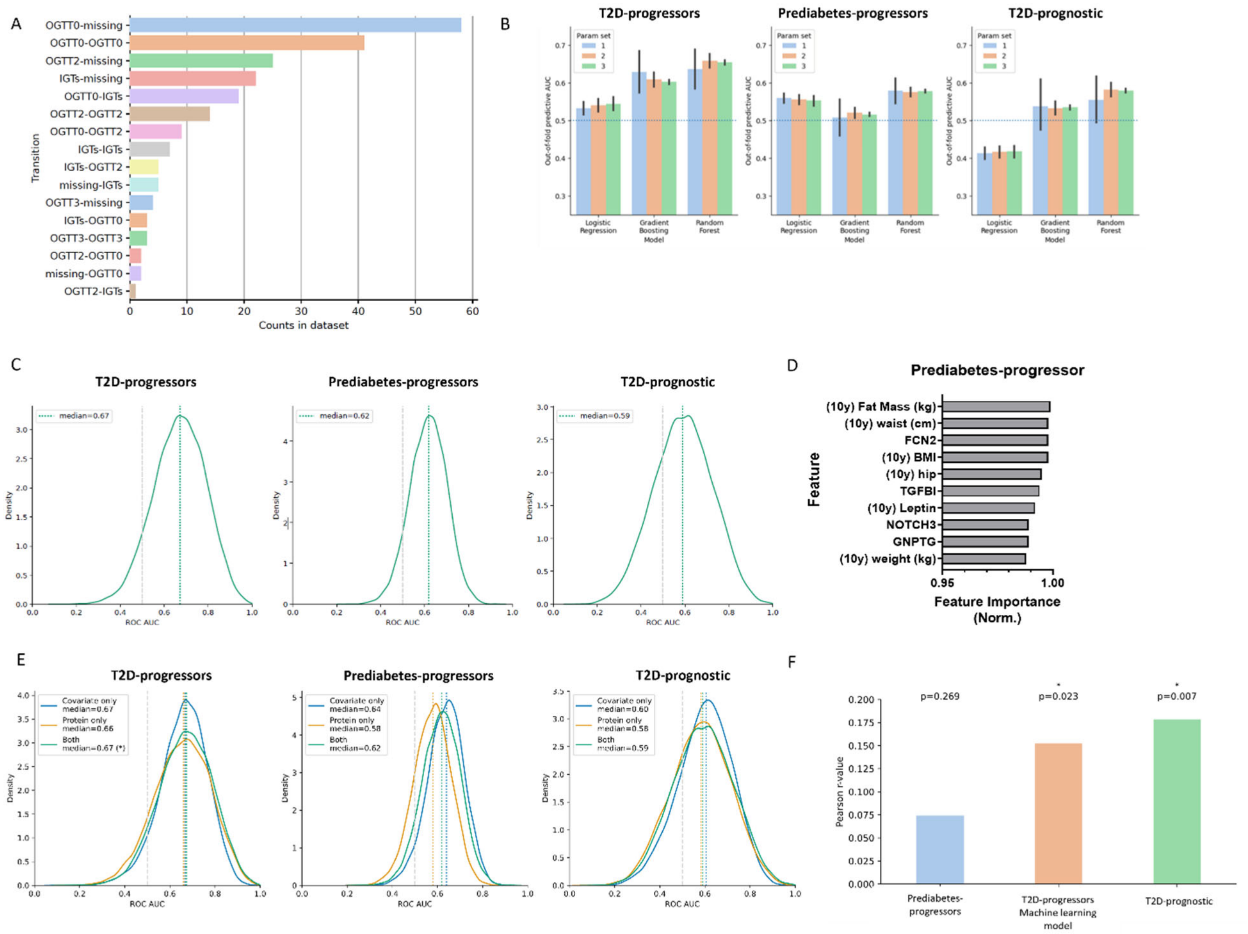

Supplementary Figure 5. Machine Learning (ML) analysis

(A) Breakdown of diabetic disease transitions from 6-year to 10-year. Oral glucose tolerance tests (OGTT) 0-OGTT2 and OGTT0-IGTs correspond to T2D-progressors and prediabetes-progressors, respectively.

(B) Estimated cross-validated AUC by three scenarios, exploring three different parameter values for each model

(C) Cross-validated predictive AUC using random forest with clinical covariates. Corresponding AUC with true positive and false positive rates are displayed in Figure 3D and 4G.

(D) Normalized feature importance of prediabetes-progressors (using permutation importance).

(E) Cross-validated predictive AUC using Random Forest with clinical covariates. (*)=significant improvement (p<0.05) over clinical covariate-only model.

(F) Summary of Pearson r-value correlating ML feature importance and -log(p-values) from UKB data, to validate the feature importance from PONCH study with UKB data.

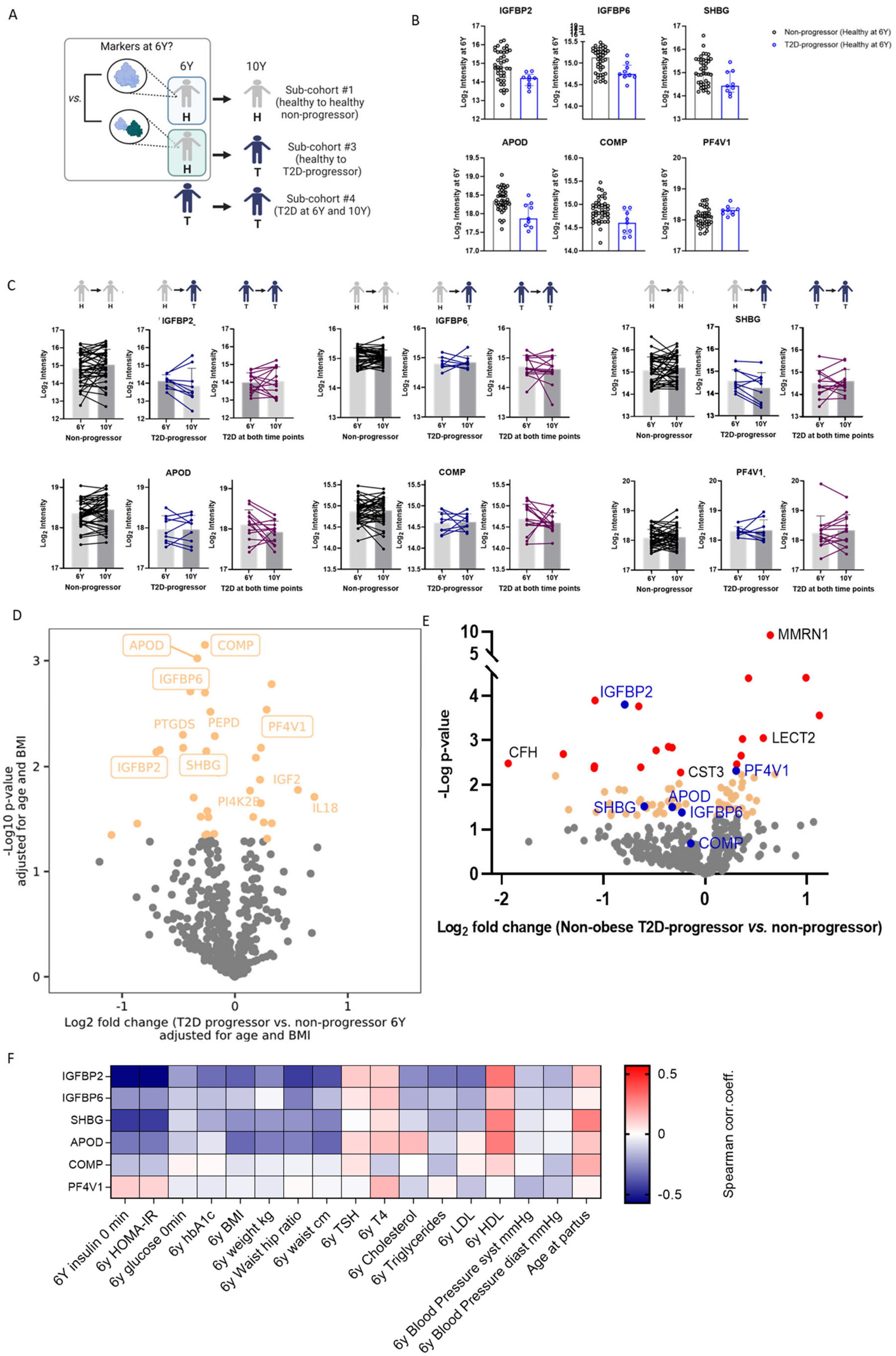
A
Markers at 6Y?
6Y
10Y
vs.
H
T
Sub-cohort #1 (healthy to healthy non-progressor)
Sub-cohort #3 (healthy to T2D-progressor)
Sub-cohort #4 (T2D at 6Y and 10Y)
B
IGFBP2
IGFBP6
SHBG
APOD
COMP
PF4V1
Log2 Intensity at 6Y
Non-progressor (Healthy at 6Y)
T2D-progressor (Healthy at 6Y)
C
Log2 Intensity
6Y
10Y
Non-progressor
T2D-progressor
T2D at both time points
D
APOD
COMP
IGFBP6
PTGDS
PEPD
PF4V1
IGFBP2
SHBG
IGF2
PI4K2B
IL18
-Log10 p-value adjusted for age and BMI
Log2 fold change (T2D progressor vs. non-progressor 6Y adjusted for age and BMI
E
MMRN1
IGFBP2
CFH
LECT2
CST3
PF4V1
APOD
SHBG
IGFBP6
COMP
-Log p-value
Log2 fold change (Non-obese T2D-progressor vs. non-progressor)
F
IGFBP2
IGFBP6
SHBG
APOD
COMP
PF4V1
6Y insulin 0 min
6y HOMA-IR
6y glucose 0min
6y hbA1c
6y BMI
6y weight kg
6y Waist hip ratio
6y waist cm
6y TSH
6y T4
6y Cholesterol
6y Triglycerides
6y LDL
6y HDL
6y Blood Pressure syst mmHg
6y Blood Pressure diast mmHg
Age at partus
Spearman corr.coeff.

Supplementary Figure 6. Differential expression of 6 protein markers

(A) Sub-cohorts for prognostic marker discovery.

(B) Expanded differential expression of 6 protein markers in healthy non-progressor *vs.* T2D-progressor participants at 6-year follow-up, when non-progressors (sub-cohort #1) and T2D-progressors (sub-cohort #3) were healthy.

(C) Protein changes within participant at 6-year *vs.* 10-year follow-up for each sub-cohort;1. Healthy non-progressors at both time points (black). 2. Healthy to T2D progressors (blue). 3. T2D at both time points (sub-cohort #4, red). Bar graphs represent mean and standard deviations of each year.

(D) Volcano plots of age and BMI adjusted analysis comparing the serum proteomes of T2D progressor (sub-cohort #3) vs. non-progressor (sub-cohort #1) at 6-year.

(E) Volcano plot displaying p-values and differential protein expression in non-obese T2D-progressor (n=5) *vs.* non-progressor at 6-year visit (n=41). Color code is based on the raw p-value <0.05 (in yellow) and FDR-corrected p-value < 0.1 (in red). The 6 prognostic markers were labeled and marked in blue and showed the same trend in non-obese T2D-progressor group.

(F) Spearman rank correlation of the abundance of the 6 candidate markers with corresponding insulin resistance of "healthy" participants at 6-year time point. Spearman correlation coefficient was color-coded.

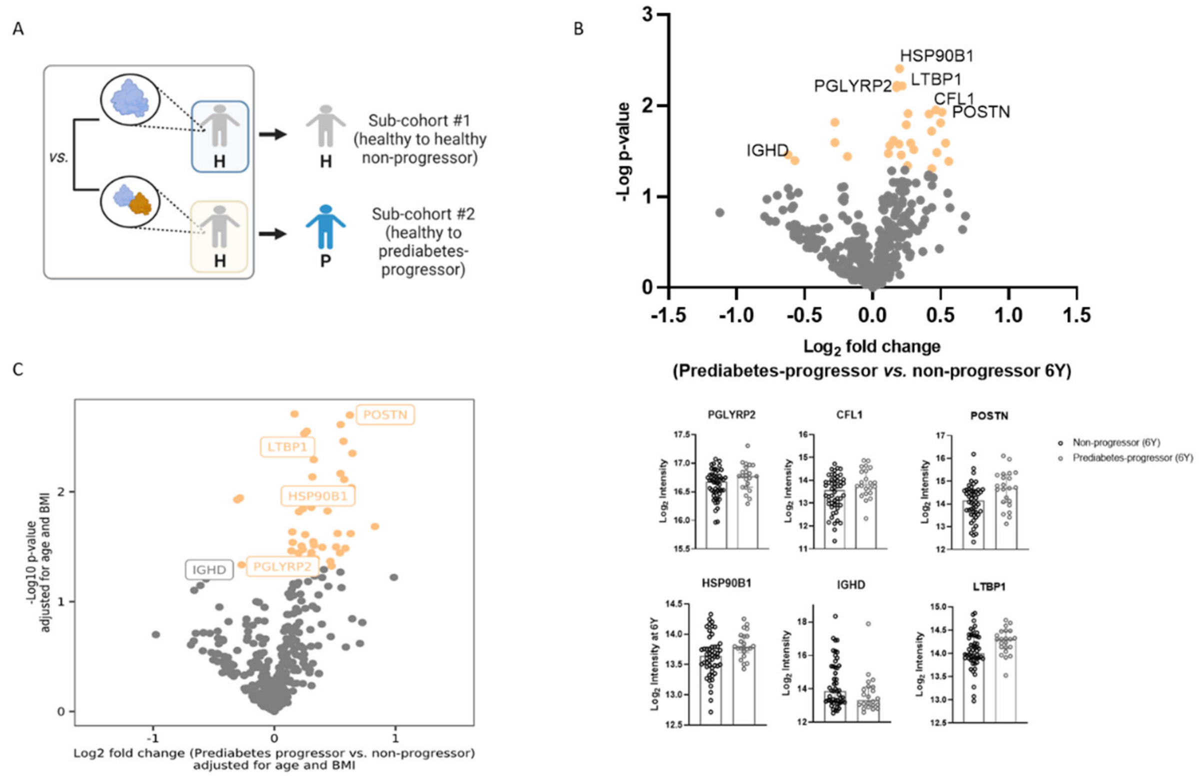

Supplementary Figure 7. Differential expression of non-progressor *vs.* progressors at 6-year visit, when their glycemic status was normal

(A) Sub-cohorts for prognostic marker discovery.

(B-C) Differential expression in non-progressor (sub-cohort #1) *vs.* prediabetes-progressor (sub-cohort #2) participants at 6-year visit, when both sub-cohorts were healthy, with (B) and without (C) age and BMI adjustment. Volcano plot displaying differential protein expression at 6-year visit (B, top). The log2 fold change in protein level is displayed on the x axis and the –log10 Welch's t -test p-value on the y axis. Color code is based on the raw p-value (p <0.05: in yellow). Individual data of the proteins where the raw p-value was lowest (B, bottom).

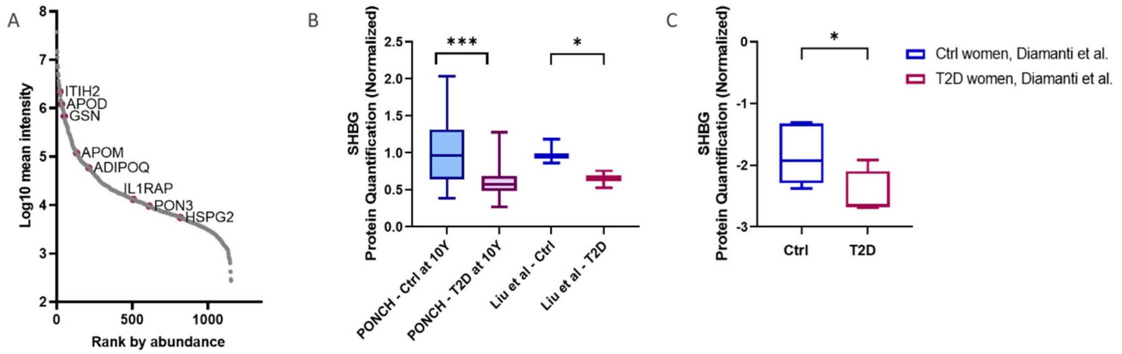

Supplementary Figure 8. Proteomic profiling of a validation cohort and replication of the findings from PONCH study in two independent analyses

(A) Proteins identified in general T2D validation cohorts were ranked according to their MS signals. Over 1300 proteins total were quantified and protein intensities spanned over five orders of magnitude. Protein examples discussed in this study labeled in mulberry.

(B-C) Comparison with two recently published studies, in Liu *et al.*, 2021(33) and Diamanti *et al.*(34). Protein quantity of SHBG (sex-hormone binding globulin) detected in Liu *et al* (B), and serum SHBG in post-menopausal non-obese women in the Diamanti *et al*. (n=7 controls, and n=4 T2D) (C). Welch t-test was performed on normalized published data.

References (for Supplemental Documents)

1. Bycroft C, Freeman C, Petkova D, Band G, Elliott LT, Sharp K, et al. The UK Biobank resource with deep phenotyping and genomic data. *Nature.* 2018;562(7726):203-9.
2. Dhindsa RS, Burren OS, Sun BB, Prins BP, Matelska D, Wheeler E, et al. Rare variant associations with plasma protein levels in the UK Biobank. *Nature.* 2023;622(7982):339-47.
3. Wang Q, Dhindsa RS, Carss K, Harper AR, Nag A, Tachmazidou I, et al. Rare variant contribution to human disease in 281,104 UK Biobank exomes. *Nature.* 2021;597(7877):527-32.
4. Middleton L, Melas I, Vasavda C, Raies A, Rozemberczki B, Dhindsa RS, et al. Phenome-wide identification of therapeutic genetic targets, leveraging knowledge graphs, graph neural networks, and UK Biobank data. *Science Advances.* 2024;10(19).
5. Garg M, Karpinski M, Matelska D, Middleton L, Burren OS, Hu F, et al. Disease prediction with multi-omics and biomarkers empowers case–control genetic discoveries in the UK Biobank. *Nature Genetics.* 2024;56(9):1821-31.
6. Tyanova S, Temu T, Sinitcyn P, Carlson A, Hein MY, Geiger T, et al. The Perseus computational platform for comprehensive analysis of (prote)omics data. *Nature Methods.* 2016;13(9):731-40.
7. Beijer K, Nowak C, Sundström J, Ärnlöv J, Fall T, and Lind L. In search of causal pathways in diabetes: a study using proteomics and genotyping data from a cross-sectional study. *Diabetologia.* 2019;62(11):1998-2006.
8. Molvin J, Pareek M, Jujic A, Melander O, Råstam L, Lindblad U, et al. Using a Targeted Proteomics Chip to Explore Pathophysiological Pathways for Incident Diabetes– The Malmö Preventive Project. *Scientific Reports.* 2019;9(1):272.
9. Liu X, Sun J, Wen X, Duan J, Xue D, Pan Y, et al. Proteome profiling of gestational diabetes mellitus at 16-18 weeks revealed by LC-MS/MS. *Journal of Clinical Laboratory Analysis.* 2020;34(9):e23424.
10. Schlitt A, Bickel C, Thumma P, Blankenberg S, Rupprecht HJ, Meyer J, et al. High Plasma Phospholipid Transfer Protein Levels as a Risk Factor for Coronary Artery Disease. *Arteriosclerosis, Thrombosis, and Vascular Biology.* 2003;23(10):1857-62.
11. Jiang X-C, Jin W, and Hussain MM. The impact of phospholipid transfer protein (PLTP) on lipoprotein metabolism. *Nutrition & Metabolism.* 2012;9(1):75.
12. Cavusoglu E, Marmur JD, Chhabra S, Chopra V, Eng C, and Jiang X-C. Relation of baseline plasma phospholipid transfer protein (PLTP) activity to left ventricular systolic dysfunction in patients referred for coronary angiography. *Atherosclerosis.* 2009;207(1):261-5.
13. van Tol A. Phospholipid transfer protein. *Current Opinion in Lipidology.* 2002;13(2):135-9.
14. Ravnsborg T, Svaneklink S, Andersen LLT, Larsen MR, Jensen DM, and Overgaard M. First-trimester proteomic profiling identifies novel predictors of gestational diabetes mellitus. *PLOS ONE.* 2019;14(3):e0214457.
15. Moin ASM, Nandakumar M, Diboun I, Al-Qaissi A, Sathyapalan T, Atkin SL, et al. Hypoglycemia-induced changes in complement pathways in type 2 diabetes. *Atherosclerosis Plus.* 2021;46:35-45.
16. Lepedda AJ, Lobina O, Rocchiccioli S, Nieddu G, Ucciferri N, De Muro P, et al. Identification of differentially expressed plasma proteins in atherosclerotic patients with type 2 diabetes. *Journal of Diabetes and its Complications.* 2016;30(5):880-6.
17. Mustaniemi S, Morin-Papunen L, Keikkala E, Öhman H, Surcel HM, Kaaja R, et al. Associations of low sex hormone-binding globulin and androgen excess in early pregnancy with fasting and post-prandial hyperglycaemia, gestational diabetes, and its severity. *Diabetes/Metabolism Research and Reviews.* 2023;39(2).

18. Buttari B, Riganò R, Palmieri L, Lo Noce C, Blankenberg S, Zeller T, et al. Sex Hormone-Binding Globulin and Its Association to Cardiovascular Risk Factors in an Italian Adult Population Cohort. *Reports.* 2022;5(1):5.
19. O'Reilly MW, Glisic M, Kumarendran B, Subramanian A, Manolopoulos KN, Tahrani AA, et al. Serum testosterone, sex hormone-binding globulin and sex-specific risk of incident type 2 diabetes in a retrospective primary care cohort. *Clinical Endocrinology.* 2019;90(1):145-54.
20. Rooney MR, Chen J, Echouffo-Tcheugui JB, Walker KA, Schlosser P, Surapaneni A, et al. Proteomic Predictors of Incident Diabetes: Results From the Atherosclerosis Risk in Communities (ARIC) Study. *Diabetes Care.* 2023;46(4):733-41.
21. Ngo D, Benson MD, Long JZ, Chen Z-Z, Wang R, Nath AK, et al. Proteomic profiling reveals biomarkers and pathways in type 2 diabetes risk. *JCI Insight.* 2021;6(5).
22. Ahmad E, Lim S, Lamptey R, Webb DR, and Davies MJ. Type 2 diabetes. *The Lancet.* 2022;400(10365):1803-20.
23. Sriboonvorakul N, Hu J, Boriboonhirunsarn D, Ng LL, and Tan BK. Proteomics Studies in Gestational Diabetes Mellitus: A Systematic Review and Meta-Analysis. *Journal of Clinical Medicine.* 2022;11(10):2737.
24. Alessi M-C, Nicaud V, Scroyen I, Lange C, Saut N, Fumeron F, et al. Association of vitronectin and plasminogen activator inhibitor-1 levels with the risk of metabolic syndrome and type 2 diabetes mellitus. *Thrombosis and Haemostasis.* 2011;106(09):416-22.
25. Zhou T, Huang L, Wang M, Chen D, Chen Z, and Jiang S-W. A Critical Review of Proteomic Studies in Gestational Diabetes Mellitus. *Journal of Diabetes Research.* 2020;2020:1-13.
26. Haywood NJ, Slater TA, Matthews CJ, and Wheatcroft SB. The insulin like growth factor and binding protein family: Novel therapeutic targets in obesity & diabetes. *Molecular Metabolism.* 2019;19:86-96.
27. Noordam R, van Heemst D, Suhre K, Krumsiek J, and Mook-Kanamori DO. Proteome-wide assessment of diabetes mellitus in Qatari identifies IGFBP-2 as a risk factor already with early glycaemic disturbances. *Archives of Biochemistry and Biophysics.* 2020;689:108476.
28. Wittenbecher C, Ouni M, Kuxhaus O, Jähnert M, Gottmann P, Teichmann A, et al. Insulin-Like Growth Factor Binding Protein 2 (IGFBP-2) and the Risk of Developing Type 2 Diabetes. *Diabetes.* 2018;68(1):188-97.
29. Anderlová K, Cinkajzlová A, Šimják P, Kloučková J, Kratochvílová H, Lacinová Z, et al. Association between gestational diabetes mellitus and bioavailability of insulin-like growth factors and role of their binding proteins. *Growth Hormone & IGF Research.* 2022;67:101511.
30. Lee J-H, Kim D-Y, Pantha R, Lee E-H, Bae J-H, Han E, et al. Identification of Pre-Diabetic Biomarkers in the Progression of Diabetes Mellitus. *Biomedicines.* 2021;10(1):72.
31. Lewandowski KC, Stojanovic N, Bienkiewicz M, Tan BK, Prelevic GM, Press M, et al. Elevated concentrations of retinol-binding protein-4 (RBP-4) in gestational diabetes mellitus: negative correlation with soluble vascular cell adhesion molecule-1 (sVCAM-1). *Gynecol Endocrinol.* 2008;24(6):300-5.
32. Chen Y, Das S, Zhuo G, and Cai H. Elevated serum levels of galectin-3 binding protein are associated with insulin resistance in non-diabetic women after menopause. *Taiwanese Journal of Obstetrics and Gynecology.* 2020;59(6):877-81.
33. Liu S, Gui Y, Wang MS, Zhang L, Xu T, Pan Y, et al. Serum integrative omics reveals the landscape of human diabetic kidney disease. *Molecular Metabolism.* 2021;54:101367.
34. Diamanti K, Cavalli M, Pereira MJ, Pan G, Castillejo-López C, Kumar C, et al. Organ-specific metabolic pathways distinguish prediabetes, type 2 diabetes, and normal tissues. *Cell Reports Medicine.* 2022;3(10):100763.
